# Reduced expression of IQGAP2 and higher expression of IQGAP3 correlates with poor prognosis in cancers

**DOI:** 10.1371/journal.pone.0186977

**Published:** 2017-10-26

**Authors:** Dinesh Kumar, Md. Khurshidul Hassan, Niharika Pattnaik, Nachiketa Mohapatra, Manjusha Dixit

**Affiliations:** 1 School of Biological Sciences, National Institute of Science Education and Research, HBNI, Odisha, India; 2 SRL Diagnostics Ltd, Odisha, India; 3 Apollo Hospitals, Odisha, India; University of North Carolina at Chapel Hill School of Medicine, UNITED STATES

## Abstract

IQGAPs is a family of proteins which comprises three members, in humans. The expression pattern and role of IQGAP1 has been well established in many cancers, whereas those of IQGAP2 and IQGAP3, have mostly remained unexplored. We used available large datasets, to explore the pan-cancer status of these two genes *in-silico*. Here we have analysed their mRNA expression and correlation with survivability in eight different cancers, including lung, breast, gastric, brain, colorectal, prostate, liver and kidney cancers and, their subtypes. The mRNA expression of IQGAP2 and IQGAP3 in individual cancers were analysed in two different publicly available databases viz. Oncomine and TCGA. The prognostic value of these genes in lung, breast and gastric cancer was analysed using Kaplan-Meier Plotter database, whereas for brain, colorectal, liver, prostate and kidney cancers, SurvExpress database was used. These results were validated by immunohistochemistry in cancer tissues (stomach, prostate, brain, colorectal). Moreover, we did IQGAP2 and IQGAP3 genomic alteration and, promoter methylation analysis using cBioportal and Wanderer web tool, respectively. Most of the cancer types (lung, breast, prostate, brain, gastric, liver, kidney and colorectal) showed increased IQGAP3 mRNA expression. In contrast, the IQGAP2 transcript levels were reduced across different cancers viz. lung, breast, gastric, liver, kidney and colorectal cancer. IQGAP2 expression correlated positively with survivability, on the contrary, IQGAP3 expression levels correlated inversely with survivability, in most of the cancers. Collectively, enhanced IQGAP3 and reduced IQGAP2 levels were frequently observed in multiple cancers with the former predicting poor survivability and the later opposite. Methylation pattern was significantly altered in most of the cancer types. We found copy no. variation and mutations in specific cancers, for IQGAP2 and IQGAP3. Our *in-vivo* (IHC) data confirmed the *in-silico* findings completely. Hence, IQGAP2 and IQGAP3 have potential to be used as prognostic markers or therapeutic targets in specific cancers.

## Introduction

IQGAPs (IQ Motif Containing GTPase Activating Proteins) are evolutionarily conserved proteins [1]. In majority of the vertebrates, three related isoforms of IQGAP protein family viz. IQGAP1, IQGAP2 and IQGAP3 are expressed. IQGAP1 is the first identified member of this family and is expressed ubiquitously [2]. The two other members IQGAP2 and IQGAP3 were identified later and their expression is limited to some organs i.e. liver, gastric, prostate, brain, lung and testis [3–7]. All these members share high identity in five conserved domains viz. calponin homology domain (CHD), poly-proline protein-protein domain (WW), IQ domain with four IQ motifs (IQ), RasGAP-related domain (GRD) and carboxy-terminal domain (RasGAP C) [8]. Multiple proteins interact to these domains and regulate diverse cellular processes, including cell migration, cell proliferation, vesicle trafficking, cytokinesis, intracellular signalling, and cytoskeletal dynamics [9]. Despite of sharing five highly identical domains, the roles of these isoforms vary significantly [1].

IQGAPs have been reported to play crucial roles in cancer progression. Multiple studies have investigated and identified IQGAP1 as an oncogene in different cancers [10]. However, only a few reports, in fewer sample size, are available which provide information regarding the expression pattern of IQGAP2 and IQGAP3 in different cancer types and their role in cancer development. Reduced expression of IQGAP2 has been found in cancers of gastric, liver, prostate and ovarian origin [11–14]. On the other hand, increased IQGAP3 expression is observed in lung, liver, breast and pancreatic cancer [15–18].

These preliminary studies indicate that apart from IQGAP1, the other two isoforms might also play crucial role in tumorigenesis. But in the absence of study, providing a comprehensive analysis of the expression pattern of these isoforms across different cancers, it is difficult to assign any prognostic or therapeutic value to either of these isoforms. In this study, we have carried out data-mining across different publicly available databases to explore expression levels of these two isoforms in different cancers (lung, breast, gastric, colorectal, brain, prostate, liver and kidney cancer), their subtypes and correlation with survivability of cancer patients.

## Methods

### Oncomine analysis

To analyse the transcript levels of IQGAP2 and IQGAP3 in different cancer subtypes, Oncomine [https://www.oncomine.org, Compendia biosciences, Ann Arbor, MI, USA] [19], an online microarray database was used. The search parameters used in this database were as follows; analysis type- cancer vs normal, data source- public, cancer types- selected specific cancer type, sample type- clinical specimen and, data type- mRNA. The studies that fulfilled the criteria, p-value ≤ 0.01, fold change ≥ 2 fold and gene rank ≤ 10%, were included in this study.

### TCGA analysis using UCSC Xena browser

TCGA is a collection of web-based tools that visualize, integrate and analyse cancer genomics and its associated clinical data. For this study, Integrin mRNA HiSeq expression data for lung, breast, colorectal, gastric, brain, prostate, liver and kidney cancer were downloaded using the latest UCSC Xena browser (http://xena.ucsc.edu/) version: 2016-08-16. Subsequently, mRNA expression of normal tissues was compared with the respective cancer tissues. Significance of difference between mean expression values of two groups, was determined by Student’s *t*-test, (unpaired, two-tailed) using GraphPad prism version 6.0. The results were considered to be significant with p-value ≤ 0.01. Furthermore, to explore the role of these genes in tumor initiation or tumor progression, comparison of IQGAP2 and IQGAP3 mRNA expression was done among different cancer stages. Data analysis was done in GraphPad prism version 6.0 and were considered significant with p-value ≤ 0.05.

### Kaplan-Meier plotter analysis

Kaplan-Meier Plotter (http://kmplot.com/analysis/) [20], a database that integrates gene expression data and clinical data, was used to analyse the prognostic value of IQGAP2 and IQGAP3 in lung, breast and gastric cancer. Till 5^th^ April 2017, the Kaplan Meier plotter had information of 54,675 genes on survival, including 5143 breast, 1816 ovarian, 2437 lung and 1065 gastric cancer patients with a mean follow-up of 69 / 40 / 49 / 33 months, respectively. We focused our analysis on overall patient survival (OS), first progression (FP), post progression survival (PPS), distance metastasis free survival (DMFS) and relapse free survival (RFS), using best JetSet probe. The hazard ratio (HR), with 95% confidence intervals and log rank p-value, was calculated in R statistical software (http://www.r-project.org) using Bioconductor packages. The HR, having p-value ≤ 0.05 was considered significant. False discovery rate (FDR) was computed to correct for multiple testing using the brainwaver library in R statistical software. FDR cut off was set at 5%. Expression level, between cancerous and normal samples, was compared using a Mann-Whitney U-test.

### SurvExpress database analysis

SurvExpress (http://bioinformatica.mty.itesm.mx:8080/Biomatec/SurvivaX.jsp) [21], is a large database that facilitates comparisons and validations of prognostic and predictive biomarkers for cancer outcomes. The survival data for five other cancers, which was not available with Kaplain Meier Plotter, was analysed with this database. Primarily, TCGA datasets were considered for analysis because of the presence of both IQGAP2 and IQGAP3 gene probes and larger sample size (>200 patient). The parameters used for survival analysis was as follows; cancer type-specific cancer name, dataset- TCGA, duplicate gene-max gene probe expression and max high risk. HR with 95% confidence interval (CI) having p-value ≤ 0.05 were considered significant.

### Somatic mutation and copy number alteration analysis

In order to find somatic mutations and copy number change of IQGAP2 and IQGAP3, the genomic data was retrieved from TCGA datasets using cBioportal (http://www.cbioportal.org/)) [22,23]. Briefly, the cancer specific TCGA datasets were selected followed by selection of mutation and CNV datatype and, IQGAP2 and IQGAP3 gene symbols, in the specified columns. On submitting the query, the software shows all types of genomic alterations including somatic mutations, copy number change, and mRNA expression, in a concise graphical summary called oncoprint. In order to understand the potential role of different mutations in terms of driver (changes protein function) or passenger (no role in fitness of a clone but associated with clonal expansion) mutation a bioinformatic tool, namely mutation assessor (link available in mutation menu), was used. The mutation was considered as pathological if the functional impact score (FIS) ranges ≥ 1.93 or neutral mutation if the FIS was <1.93. The correlation between copy number changes with mRNA expression was analysed using ‘mRNA vs copy number option’, in order to understand the effect of copy number change in mRNA expression.

### Methylation status analysis

In order to see the effect of epigenetic modification i.e. DNA methylation, we analysed the methylation pattern of IQGAP2 and IQGAP3 in promoter region (mainly CpG islands) of normal vs cancer specimens using Wanderer web tool (http://maplab.imppc.org/wanderer/doc.html)) [24]. This tool allows real time access and visualization of gene expression and DNA methylation profiles from TCGA. The differences in methylation pattern in promoter regions (tumor vs. normal) of the two IQGAP isoforms, was analysed for specific TCGA cancer dataset and considered significant at p-value ≤ 0.05. Pearson correlation method was used to find out the correlation between methylation and gene expression of IQGAP2 and IQGAP3. The correlation was examined between probes showing significant change at promoter region and mRNA expression of single exon. The correlation was considered strong if the Pearson’s correlation coefficient (r) was 0.5 to 1.0 or -0.5 to-1.0, moderate if r = 0.3 to 0.5 or -0.3 to-0.5, or weak if r = 0.1 to 0.3 or -0.1 to -0.3.

### Immunohistochemistry

For IQGAP2 and IQGAP3 expression analysis in tumor and normal tissue, archival formalin-fixed paraffin-embedded (FFPE) specimens of 53 colorectal cancer, 47 stomach cancer, 49 brain cancer, 32 prostate cancer and 19 benign prostate hyperplasia (BHP), were obtained from the Department of Pathology of Apollo hospital, Bhubaneswar and SRL Diagnostics Ltd, Bhubaneswar. All of the tumor samples were again confirmed by two independent pathologists (N.P. and N.M.) in Clinical Pathology Department of the hospitals. The study was approved by the Institutional Ethics Committee, NISER, Bhubaneswar (protocol no. NISER/IEC/2016-01). The need for informed consent was waived by Institutional Ethics Committee, National Institute of Science Education and Research (NISER), Bhubaneswar, India, based on waiver of consent policy of Ethical Guidelines for Biomedical Research on Human Participants, ICMR, 2006 (http://icmr.nic.in/ethical_guidelines.pdf).

For immunohistochemistry, 5 μm thin sections of prostate, brain, colorectal and stomach cancer and unmatched control, were cut from FFPE biopsy specimens. Sections were placed on poly L-lysine coated glass slides and immunostained with Envision Flex mini kit (Dako). Briefly, section were deparaffinized, followed by rehydration. Heat induced (microwave) epitope retrieval was done, in citrate retrieval solution low pH. To stop endogenous peroxidase activity, the specimens were immersed for 15 minutes in Envision Peroxidase Blocker (Dako). The tissue section was treated with primary antibody for IQGAP2 (Abcam) at 1:100 dilution or for IQGAP3 (Sigma) at 1:500 dilution, followed by 1 hour incubation in a humidified chamber, at room temperature. Sections were washed and incubated with Envision Flex HRP secondary antibody (Dako). Liquid DAB Substrate was used for staining. The sections were counterstained with hematoxylin, dehydrated, and cover-slipped with permanent media and observed independently by two pathologists (N.P. and N.M.) for the unbiased scoring of the markers.

The scoring was categorized as weak, moderate and strong according to the IRS scoring system. The reaction was considered as positive when brown signals appeared in the cell cytoplasm, nucleus or membrane. IRS score was determined by multiplying the score of staining intensity and score of positive area. The score of staining intensity was kept in range of 0 to 3 based on: 0 for no staining, 1 for weak staining, 2 for moderate staining and, 3 for strong staining. The scores for cell positivity were given in the range of 1 to 4, based on: 1 for positive staining in 0–25% of tumor cells, 2 for positive staining in 25–50% of tumor cells, 3 for positive staining in 51–75% of tumor cells and, 4 for positive staining in 75–100% of tumor cells. Further, for statistical analyses the IRS scores of 0–4 were treated as weak staining, scores of 5–8 as moderate staining and, scores of 9–12 as intense staining.

## Results

### IQGAP2 and IQGAP3 expression in different cancers

**Fig 1:**
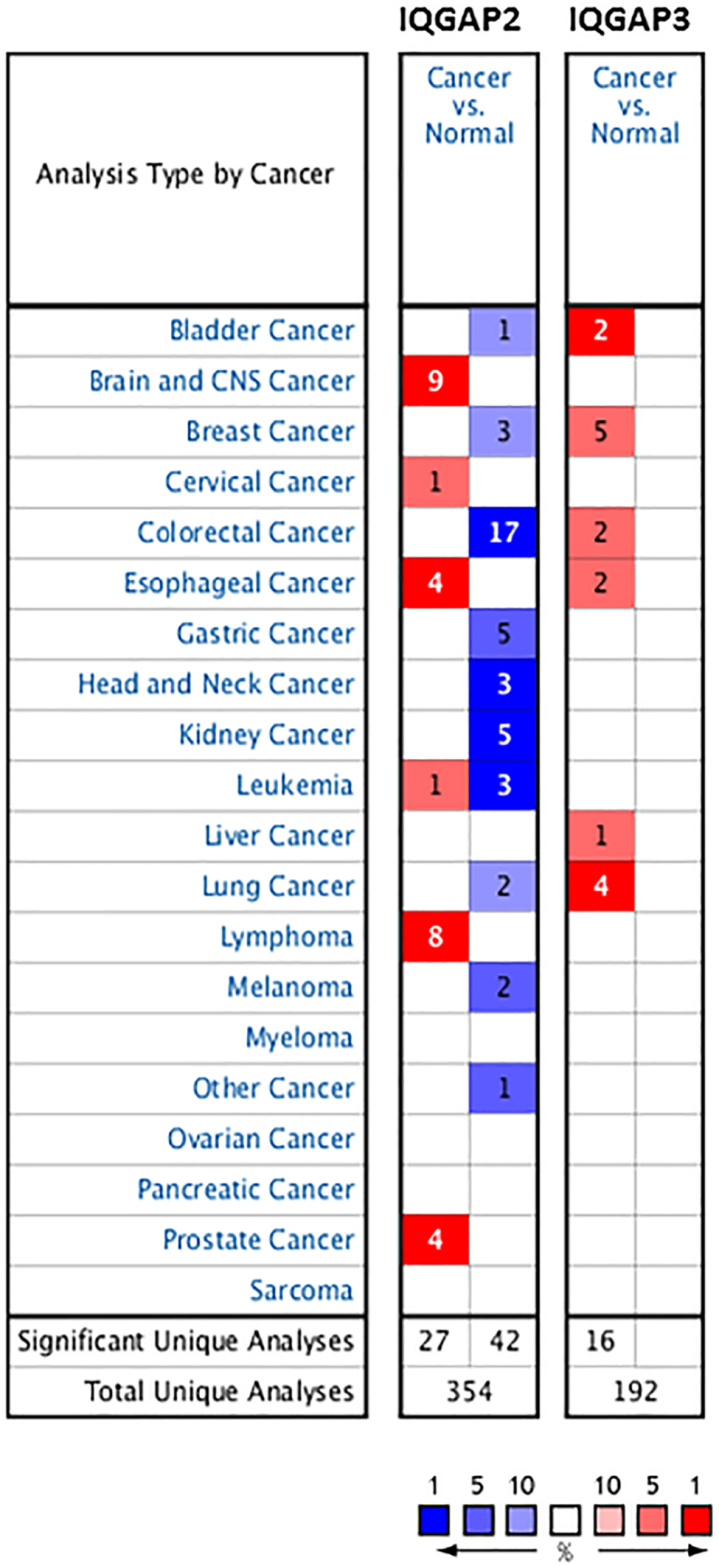
The mRNA expression patterns of IQGAP2 and IQGAP3 in cancers. The mRNA expression difference, between tumors and normal tissues, were analysed in Oncomine database with thresholds of p-value ≤ 0.01, fold change ≥ 2 fold, gene rank ≤ 10%. The numbers in the colored cell represent the number of analyses meeting these thresholds. The red cells indicate increased mRNA expression whereas blue cells indicate reduced mRNA expression in tumor tissues, than in normal tissues. The color depth represents the gene rank, darker color shows better percentile for the analysis within the colored cell.

Oncomine was used to find out differences in mRNA expression of IQGAP2 and IQGAP3, between tumor and normal tissue, in multiple cancers (Fig 1). The database contained a total of 354 and 192 unique analyses for IQGAP2 and IQGAP3, respectively. As per the gene summary view, a total of 27 analyses comprising brain and CNS cancer, cervical cancer, esophageal cancer, leukaemia, lymphoma and prostate cancer, showed statistically significant higher IQGAP2 mRNA expression levels in tumor, while 42 analyses including bladder cancer, breast cancer, colorectal cancer, gastric cancer, head and neck cancer, kidney cancer, leukamia, lung cancer and melanoma showed reduced expression. For IQGAP3, all 16 analyses, including bladder cancer, breast cancer, colorectal cancer, esophaseal cancer, liver cancer and lung cancer, showed significantly higher mRNA expression in cancer tissues, compared to normal tissue.

### Lung cancer

There are three main types of lung cancer; non-small cell lung cancer (NSCLC), small cell lung cancer (SCLC) and lung carcinoid tumor. Among these three, NSCLC is the most common type of lung cancer (85%) followed by SCLC (10–15%) and lung carcinoid tumor (5%). NSCLC can be divided into three main subtypes: adenocarcinoma (AC, 40% of lung cancers), lung squamous cell carcinoma (LUSC, 25–30% of lung cancers), and large cell lung carcinoma, (LCLC, 10% of lung cancers). Mixed pattern of adenocarcinomas are more common than tumors showing a single pattern (e.g. acinar, papillary, bronchioloalveolar, and solid adenocarcinoma with mucin formation).

#### IQGAP2 and IQGAP3 expression in lung cancer

**Fig 2:**
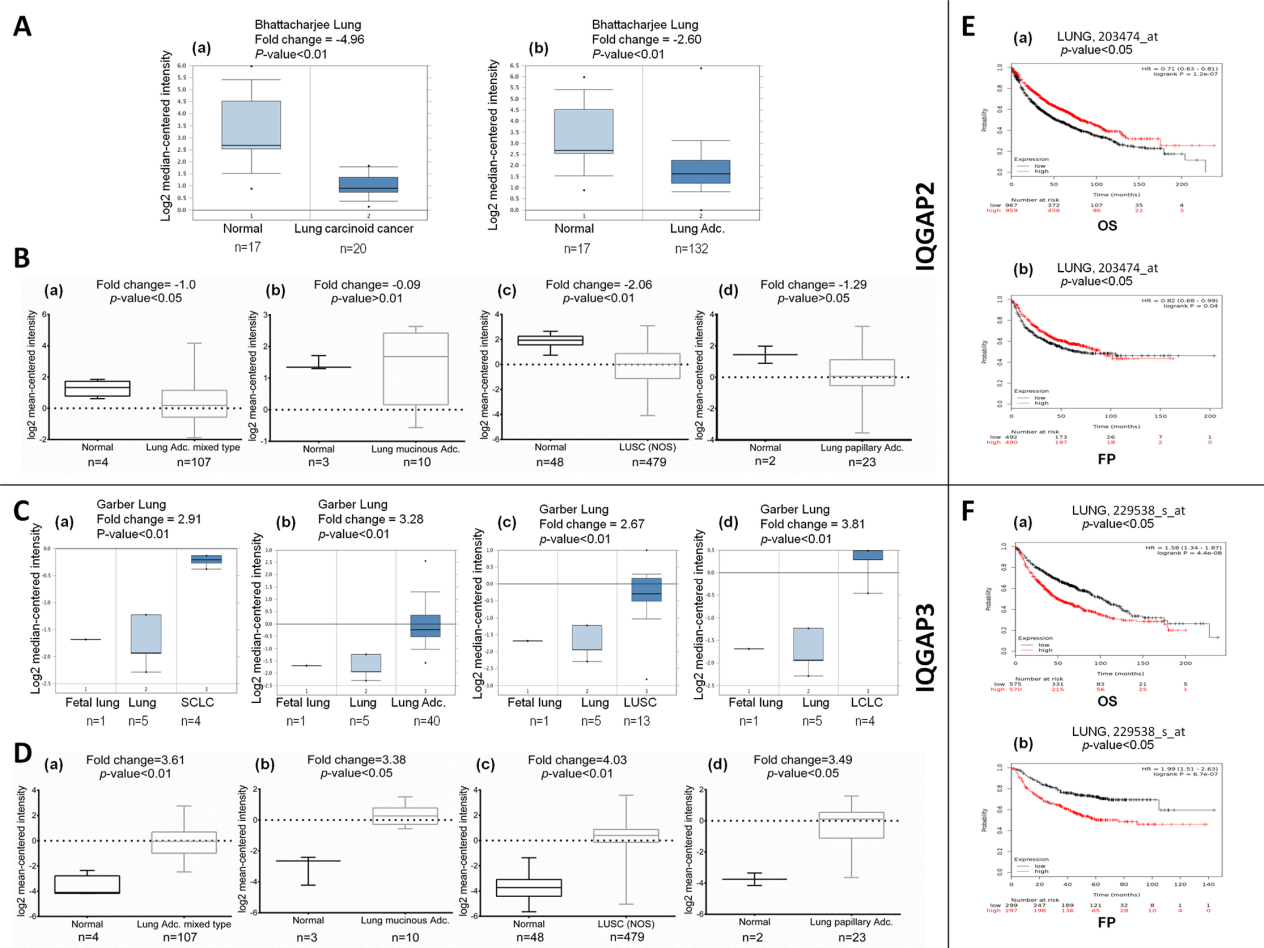
The mRNA expression of IQGAP2 and IQGAP3 in lung cancer subtypes and its correlation with survival of the patients. (A) and (B): Box and whisker plots showing IQGAP2 mRNA expression in lung cancer subtypes in Oncomine and TCGA database, respectively. X-axis of the plot represents normal vs cancer group, Y-axis represents mRNA expression in log2 median/mean centred intensity. The line in the middle represents the median value. (C) and (D): Box and whisker plots showing IQGAP3 mRNA expression in lung cancer subtypes in Oncomine and TCGA database, respectively. X-axis of the plot represents normal vs cancer group, Y-axis represents mRNA expression in log2 median/mean centred intensity. The line in the middle represents the median value. Differences were examined statistically by two-tailed student’s t-test. (E) and (F): Kaplan-Meier plots showing overall survival (OS) and first progression (FP) in lung cancer and its association with IQGAP2 and IQGAP3 expression levels, respectively. In this graph X-axis denotes, number of patients at risk at specific time (in months) and Y-axis denotes the probability of survival. In red, patients with expression above the median value and in black, patients with expressions below the median value, have been represented. The hazard ratio (HR), 95% CI, p-value ≤ 0.05 were considered significant.

Oncomine analysis of cancer vs. normal tissue in different datasets showed altered expression of IQGAP2 and IQGAP3 in different subtypes of lung cancer (Fig 2). In Bhattacharjee lung dataset [25], the mRNA level of IQGAP2 was significantly decreased in lung carcinoid tumor (fold change = -4.95) and lung adenocarcinoma (fold change = -2.60) (Fig 2A). On the other hand, in the analysis of the expression levels of IQGAP3 in Garber dataset [26], the subtypes of lung cancer revealed significantly increased mRNA levels of IQGAP3 in lung adenocarcinoma (fold change = 3.2), in SCLC (fold change = 2.91), in LCLC (fold change = 3.81) and, in LUSC (fold change = 2.67) (Fig 2C). Furthermore, analysis of lung cancer TCGA datasets viz. TCGA-LUNG, showed reduced expression of IQGAP2 in lung adenocarcinoma mixed type (fold change = -1.0) and in lung squamous cell carcinoma (fold change = -2.06), but not in lung papillary adenocarcinoma and mucinous cell lung carcinoma (Fig 2B). On the contrary, increased expression of IQGAP3 was observed in all of these lung cancer subtypes (Fig 2D).

Kaplan-Meier plotter analysis was carried out to determine the correlation between expression levels of both IQGAP isoforms, with FP, OS and PPS of lung cancer patients. The data from the respective probes (probe IDs listed in S1 Table) showed reduced OS and FP, for patients with low IQGAP2 expression (Fig 2E). Likewise, PPS was also diminished with reduced IQGAP2 expression in lung cancer patients (S1 Table). In contrast, patient’s survivability was inversely related with IQGAP3 expression. The OS of the lung cancer patients was less with increased IQGAP3 transcript level. Additionally, higher IQGAP3 expression led to reduced FP and PPS in lung cancer patients (Fig 2F).

### Breast cancer

Breast cancer can be broadly categorised into ductal carcinoma *in situ*, invasive or infiltrating ductal carcinoma and invasive lobular carcinoma. Invasive breast cancers can be further sub-divided into groups based on the presence or absence of hormone receptors viz. estrogen receptor (ER), progesterone receptor (PR) and epidermal growth factor receptor 2 (HER2). Based on these molecular markers, breast cancer is classified into hormone receptor-positive, hormone receptor-negative, HER2 positive, HER2 negative, triple-negative and triple positive. The correlation of a particular oncogene or tumor suppressor gene with a particular marker plays a vital role in predicting its prognostic value with regards to responsiveness to a particular treatment regime.

#### IQGAP2 and IQGAP3 expression in breast cancer

**Fig 3:**
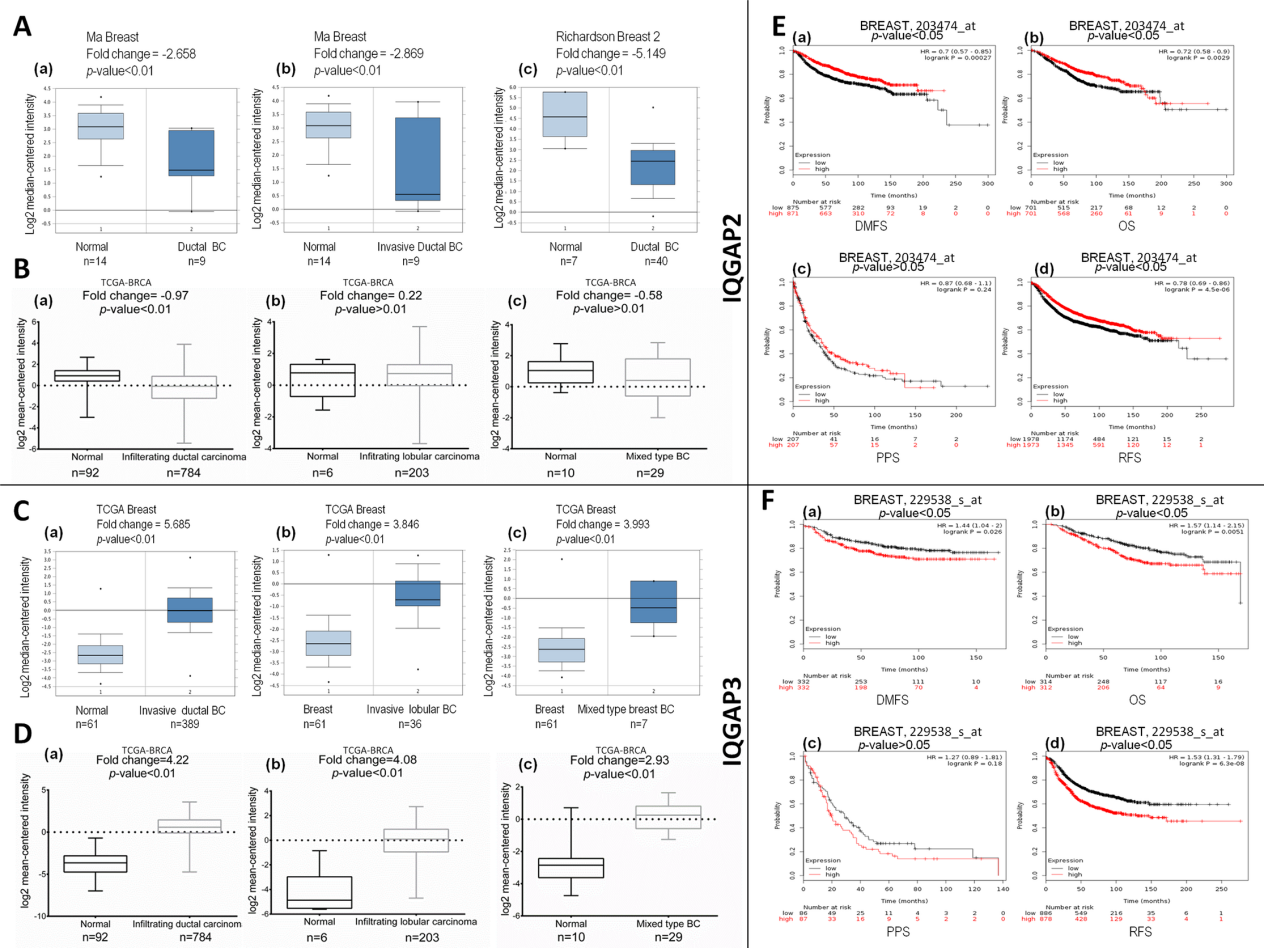
The mRNA expression of IQGAP2 and IQGAP3 in breast cancer subtypes and correlation with survival of the patients. (A) and (B): IQGAP2 mRNA expression as seen in different breast cancer subtypes in Oncomine and TCGA database, respectively. (C) and (D): IQGAP3 mRNA expression as seen in different breast cancer subtypes in Oncomine and TCGA database, respectively. X-axis represents normal vs. cancer group; Y-axis represents mRNA expression in log2 median/mean centred intensity (median value depicted as middle line in each box-whisker plot). Two-tailed Student’s t-test was used to ascertain statistically significant difference between the groups. (E) and (F): Kaplan-Meier plots for distance metastasis free survival (DMFS), overall patient survival (OS), post progression survival (PPS), and relapse free survival (RFS), in breast cancer patients with IQGAP2 and IQGAP3 expression, respectively. Here X-axis denotes number of patients at risk at specific time (in months) and Y-axis shows the probability of survival. Red line represents patients with expression above the median value and in black, patients with expressions below the median value have been represented. The hazard ratio (HR), 95% CI, p-value ≤ 0.05 were considered significant.

A significant change was observed in transcript levels of IQGAP2 and IQGAP3 in breast cancer compared to normal tissue, in different datasets of Oncomine. In Ma Breast dataset [27], reduced IQGAP2 expression was observed in ductal breast carcinoma *in situ* (fold change = -2.65) and in invasive ductal breast carcinoma (fold change = -2.86) (Fig 3A). Likewise, the TCGA dataset analysis for breast cancer via Xena browser also revealed reduced transcript levels of IQGAP2 in infiltrating ductal carcinoma (fold change = -0.97). However, no significant change was observed in infiltrating lobular and mixed lobular and ductal breast carcinoma (Fig 3B). In contrast, the expression of IQGAP3 was high across different breast cancer subtypes in TCGA dataset (version: 2011/09/02) analysed by Oncomine in invasive ductal breast carcinoma (fold change = 5.68), invasive lobular breast carcinoma (fold change = 3.84) and, mixed lobular and ductal breast carcinoma (fold change = 3.99, Fig 3C). In unison with the Oncomine database, the expression of IQGAP3 was significantly increased (TCGA dataset, version: 2016-08-16) in invasive ductal breast carcinoma (fold change = 4.22), invasive lobular breast carcinoma (fold change = 4.08) and, mixed lobular and ductal breast carcinoma (fold change = 2.93) (Fig 3D).

Kaplan-Meier analysis showed that in breast cancer, higher levels of IQGAP3 expression and lower levels of IQGAP2 expression correlate with poor survival of the patient. The cases with reduced IQGAP2 expression showed poor OS. Prolonged RFS was observed in patients with higher expression of IQGAP2. DMFS was longer in cases with elevated IQGAP2 expression. PPS extended in cases with higher IQGAP2 expression (Fig 3E). An opposite trend was observed with IQGAP3 wherein poor OS of patients, more frequent relapses (low RFS) and metastatic events (low DMFS) were significantly associated with higher transcript levels of IQGAP3 in breast cancer patients. PPS was also less in patients having higher IQGAP3 expression (Fig 3F). In brief, the survival of the patients decreased with low expression of IQGAP2 and with higher expression of IQGAP3.

### Gastric cancer

As per the Lauren’s classification of stomach adenocarcinoma, gastric cancer has two subtypes, intestinal type gastric adenocarcinoma and diffuse type gastric adenocarcinoma. A third type of gastric adenocarcinoma has also been introduced in this system of classification i.e. mixed type gastric adenocarcinoma, made up of both the intestinal and diffuse type gastric adenocarcinoma.

#### IQGAP2 and IQGAP3 expression in gastric cancer

**Fig 4:**
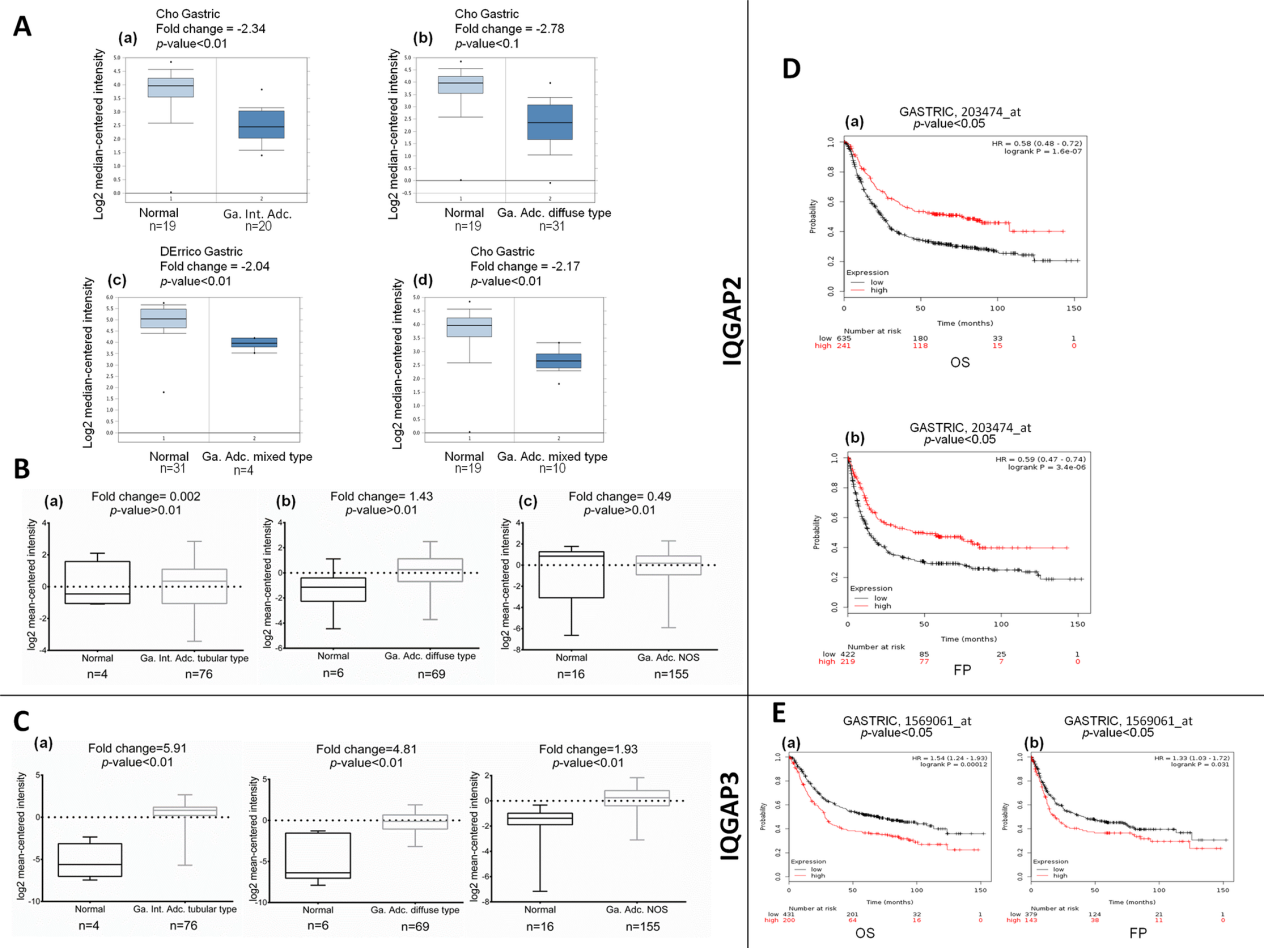
The mRNA expression of IQGAP2 and IQGAP3 in gastric cancer subtypes and correlation with survival of the patients. (A) and (B): IQGAP2 mRNA expression observed in gastric cancer subtypes as per Oncomine and TCGA database, respectively. (C): IQGAP3 mRNA expression representation as seen in TCGA database for different gastric cancer subtypes. X-axis of the plot denotes normal vs. cancer group; Y-axis represents mRNA expression in log2 median/mean centred intensity. Median value is denoted by middle line. Statistical differences were examined by two-tailed student’s t-test. (D) and (E): Kaplan-Meier plots depicting overall survival (OS) and first progression (FP) in gastric cancer with IQGAP2 and IQGAP3, respectively. In this graph, X-axis denotes number of patients at risk at specific time (in months) and Y-axis denotes the probability of survival. Red line indicates patients with expression above the median and black one represents patients with expressions below the median. The hazard ratio (HR), 95% CI, p-value ≤ 0.05 were considered significant.

In Oncomine database, IQGAP2 mRNA expression was found to be significantly decreased in different human gastric cancer subtypes, compared to normal tissues. IQGAP2 transcript levels were down regulated in gastric mixed adenocarcinoma by more than two-folds, in both DErrico Gastric [28] and Cho Gastric datasets [29] (Fig 4A). However, analysis with TCGA datasets did not show a significant change in IQGAP2 mRNA expression in any of the gastric cancer subtypes (Fig 4B). On the other hand, with the threshold criteria we had set in Oncomine database analysis, none of the studies revealed significant change in IQGAP3 mRNA levels. However, TCGA database showed increased IQGAP3 mRNA expression in gastric intestinal adenocarcinoma (fold change = 3.25), diffuse gastric adenocarcinoma (fold change = 2.51) and gastric mixed adenocarcinoma (fold change of 2.43) (Fig 4C).

The Kaplain-Meier analysis revealed significant correlation of IQGAP2 and IQGAP3 expression with the survival of the gastric cancer patients. OS and FP probability were poor in patients with lower expression of IQGAP2 (Fig 4D). In case of IQGAP3, opposite results were obtained upon analysis of two different datasets. First dataset (229538_s_at) showed a poor OS and reduced probability of FP of the patient with low IQGAP3 expression (S1 Table) whereas; second one (dataset 1569061_at) showed better OS and enhanced probability of FP with low IQGAP3 expression (Fig 4E).

### Colorectal cancer

Colorectal cancer, also called bowel cancer, arises from colon or rectum. Colorectal cancer comprises colon and rectal adenocarcinoma, leiomyosarcoma, lymphoma, melanoma, and neuroendocrine tumors. The most common, adenocarcinoma (95%) can be further sub-classified into signet ring cell adenocarcinoma and mucinous adenocarcinoma.

#### IQGAP2 and IQGAP3 expression in colorectal cancer

**Fig 5:**
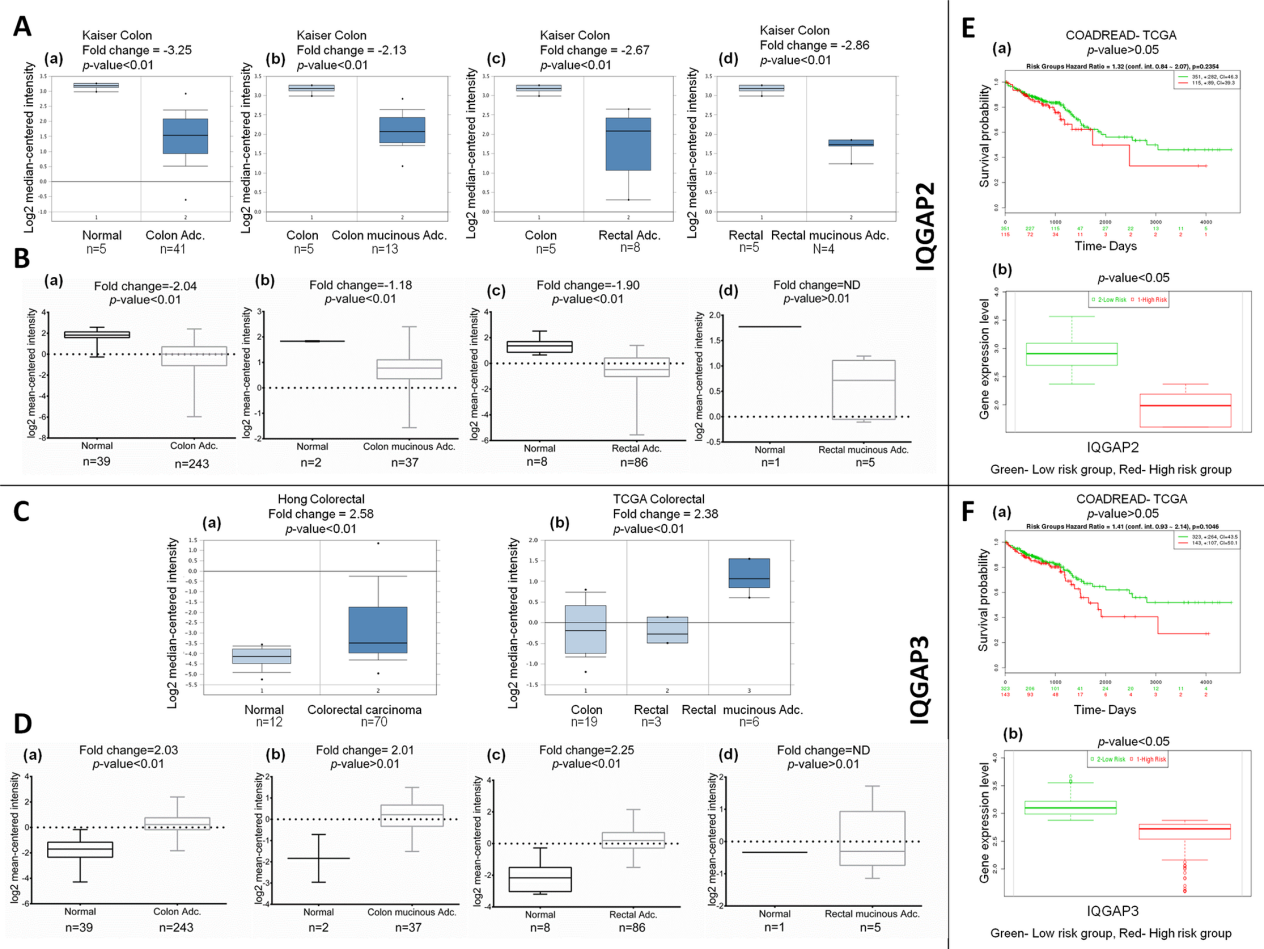
The mRNA expression of IQGAP2 and IQGAP3 in colorectal cancer subtypes and correlation with survival of the patients. (A) and (B): Box-whisker plots showing IQGAP2 mRNA expression in different colorectal cancer subtypes in Oncomine and TCGA database, respectively. (C) and (D): Oncomine and TCGA databases, respectively, showing IQGAP3 mRNA expression in different colorectal cancer subtypes. X-axis denotes normal vs. cancer group while Y-axis represents mRNA expression in log2 median/mean centred intensity. Median is represented by middle line in the box plots. Two-tailed Student’s t-test was used for ascertaining statistical difference between the groups. (E) and (F): SurvExpress database showing overall patient survival (OS) in colorectal cancer with IQGAP2 and IQGAP3 expression, respectively. E(a) and F(a) show Kaplan Meier plot of OS in gastric cancer patients with IQGAP2 and IQGAP3 expression, respectively. Here on the top, p-value and 95% CI has been shown for selected data. Survival risk curves are shown for each group; low and high risk groups are shown in green and red colors respectively. The X-axis represents the time (days) to event. The number of samples, not presenting the event at the matching time, has been shown in rows with corresponding color. E(b) and F(b) show box plot of IQGAP2 and IQGAP3 gene along with risk groups, obtained in the analysis. The X-axis shows each gene and p-value for the mean expression differences, between risk groups. The Y-axis shows the expression levels.

Oncomine database search for colorectal cancer showed 17 significant unique analyses with reduced IQGAP2 expression and two such unique analyses for IQGAP3 with increased mRNA expression (Fig 1). Reduced transcript levels of IQGAP2 were reported in colon adenocarcinoma (fold change = -3.25), colon mucinous adenocarcinoma (fold change = -2.13), rectal adenocarcinoma (fold change = -2.67) and rectal mucinous adenocarcinoma (fold change = -2.86), in Kaiser Colon dataset [30] (Fig 5A) and, in colon and rectal adenocarcinoma in other cancer datasets listed in S2 Table. TCGA colorectal cancer datasets viz. TCGA-COADREAD analysis showed low IQGAP2 mRNA expression in colon adenocarcinoma (fold change = -2.04), colon mucinous adenocarcinoma (fold change = -1.18) and rectal adenocarcinoma (fold change = -1.90) but no change in rectal mucinous adenocarcinoma (Fig 5B). In contrast to IQGAP2, significantly higher expression of IQGAP3 was found in rectal mucinous adenocarcinoma (fold change = 2.38) and colorectal carcinoma (fold change = 2.58), compared to the normal tissue (Fig 5C). Likewise, in TCGA datasets for colorectal cancer, IQGAP3 expression was found to be higher in colon adenocarcinoma (fold change = 2.03) and rectal adenocarcinoma (fold change = -2.25) but, not in colon mucinous and rectal mucinous adenocarcinoma (Fig 5D).

In the absence of availability of any survival data for colorectal cancer in Kaplain Meier plotter, SurvExpress database was used to ascertain the prognostic value of IQGAP2 and IQGAP3 in this cancer. No significant correlation was observed in either of the two TCGA datasets (COAD-TCGA and COADREAD-TCGA), between OS and either IQGAP2 or IQGAP3 mRNA expression, in colorectal cancer patients (Fig 5Ea and 5Fa) (S1 Table). However, the gene expression values of IQGAP2 were lower in high-risk group compared to the low-risk group (Fig 5Eb). On the contrary, IQGAP3 expression was increased in high-risk group (Fig 5Fb).

### Brain and CNS cancer

Brain cancer is primarily classified in primary and secondary. Primary brain tumor originates in the brain itself whereas secondary tumors are those which have originated in other organs. The primary brain tumor can further be classified into benign and malignant. Nearly 80% of the malignant primary tumors are collectively called as gliomas (because of glial cell origin). Glioma can then be further classified into different sub-types depending on the type of glial cell i.e. astrocytoma, oligodendroglioma, ependimomas and mixed glioma. Additionally, astrocytoma are classified into pilocytic astrocytoma, diffuse astrocytoma, anaplastic astrocytoma and glioblastoma multiforme (GBM), based on the grade of the tumor.

#### IQGAP2 and IQGAP3 expression in brain and CNS cancer

**Fig 6:**
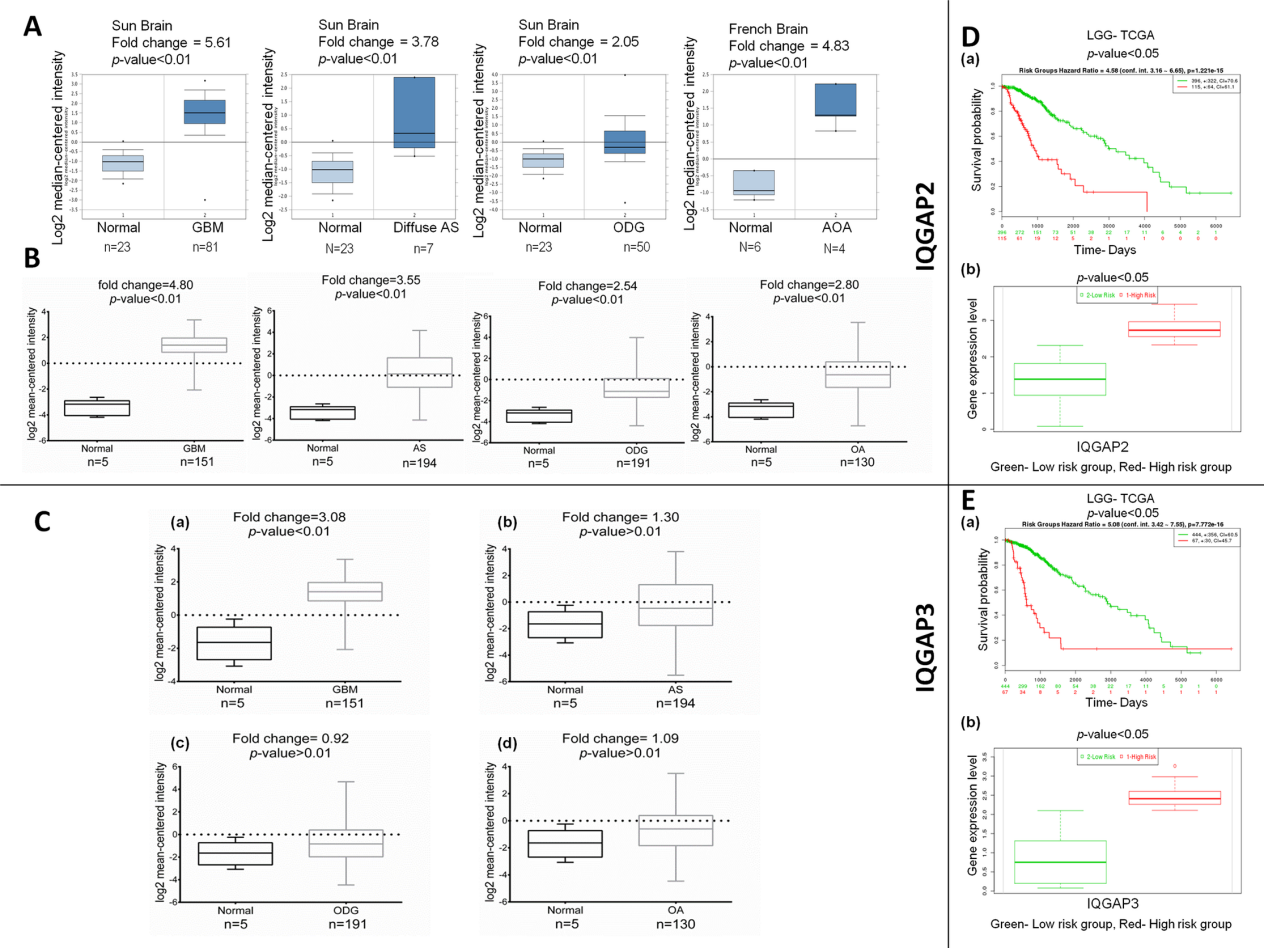
The mRNA expression of IQGAP2 and IQGAP3 in brain cancer subtypes and their correlation with survival of the patients. (A) and (B): IQGAP2 mRNA expression in Oncomine and TCGA database, respectively in different brain cancer subtypes, represented as box whisker plots (C): IQGAP3 mRNA expression from TCGA database in different brain cancer subtypes represented as box-whisker plots. X-axis of the plot represents normal vs. cancer group; Y-axis represents mRNA expression in log2 median/mean centred intensity. Line in the middle represents the median value. Differences in mean, were examined statistically by two-tailed student’s t-test. (D) and (E): SurvExpress database showing overall patient survival (OS) in brain cancer with IQGAP2 and IQGAP3 expression, respectively. Figs (Da) and (Ea) showing Kaplan Meier plot of OS of gastric brain cancer patients with IQGAP2 and IQGAP3 expression respectively. Here at the top, p-value and 95% CI have been shown with selected data. Survival risk curves are shown for each group; low and high risk groups are shown in green and red colors respectively. The X-axis represents the time (days) to event. The number of samples not presenting the event at the matching time have been shown in rows with corresponding color. Figs (Db) and (Eb), Box plot of IQGAP2 and IQGAP3 by risk groups. The gene expression of each gene is plotted along risk groups obtained in the analysis. The X-axis shows each gene and a p-value of the expression difference between risk groups. The p-value is obtained from a t-test for two risk groups. Expression levels have been shown in the Y-axis.

In brain cancer, a significant over expression of IQGAP2 was observed in different independent datasets of Oncomine. The expression levels of IQGAP2 were elevated in glioblastoma (GMB), in Sun brain [31], Murat brain [32] and, in TCGA brain dataset (S3 Table). Similarly, the mRNA expression of IQGAP2 was high in diffuse astrocytoma (fold change = 3.78), oligodendroglioma (fold change = 2.05), anaplastic oligoastrocytoma (fold change = 4.83) (Fig 6A) and anaplastic oligodendroglioma (fold change = 2.77) (S3 Table). The TCGA brain cancer datasets (GBM, LGG and GBMLGG) also showed higher expression of IQGAP2 in glioblastoma (fold change = 4.80), astrocytoma (fold change = 3.55), oligodendroglioma (fold change = 2.54) and oligoastrocytoma (fold change = 2.80) (Fig 6B). No unique analysis, in Oncomine database, fulfilled the thresholds of inclusion criteria for the IQGAP3 expression. Nonetheless, the TCGA database analysis showed higher expression of IQGAP3 in glioblastoma (fold change = 3.08) but not in other subtypes of brain cancer (Fig 6C).

LGG-TCGA survival analysis from SurvExpress database of the brain revealed poor survival of the patients with higher expression of both IQGAP2 (Fig 6D) and IQGAP3 (Fig 6E). A similar trend was obtained from survival analysis of GBMLGG-TCGA dataset (S1 Table).

### Prostate cancer

Prostate cancer has various histological subtypes but the most common type is adenocarcinoma (about 90%), which is further classified into atrophic, colloid and signet ring carcinoma. The remaining 10% of all prostate cancers include squamous cell cancer, sarcomas, carcinoid, transitional cell cancer, sarcomatoid cancers and small cell cancer.

#### IQGAP2 and IQGAP3 expression in prostate cancer

An increase in mRNA expression of IQGAP2 was found in prostate cancer, compared to the normal tissue in four datasets of Oncomine database, but the prostate cancer TCGA dataset (PRAD-TCGA) analysis from Xena browser did not show any significant difference in IQGAP2 expression. The mRNA expression of IQGAP3 did not cross the set threshold level of inclusion criteria in Oncomine database, but analysis with PRAD-TCGA dataset showed increase in its expression (fold change = 1.93) in prostate cancer tissues, compared to the normal (S4 Table).

The prostate cancer survival data analysis in TCGA dataset (PRAD-TCGA) from SurvExpress, showed poor survival of the patient with increased IQGAP3 mRNA expression (S1B Fig) but no effect of IQGAP2 expression on overall survivability of the prostate cancer patients was observed (S1A Fig).

### Liver cancer

There are two subtypes of liver cancer, based on the origin of the cancer. Primary liver cancer starts in the liver itself and can be categorised into hepatocellular cancer (HCC), intrahepatic cholangiocarcinoma, angiosarcoma and hemangiosarcoma. In secondary type, tumor cells from some other organ in the body, such as the breast, colon, stomach, or lung reach liver by metastasis and form cancer.

#### IQGAP2 and IQGAP3 expression in liver cancer

Oncomine analysis with the set thresholds did not show any studies with significantly altered IQGAP2. In TCGA database, the liver cancer TCGA dataset (LIHC-TCGA) showed low expression of IQGAP2 (fold change = -1.02) and high expression of IQGAP3 (fold change = 4.19) in liver cancer tissues, compared to the normal tissues (S4 Table).

A poor OS was observed in patients showing low IQGAP2 expression (S1C and S1D Fig) and high IQGAP3 expression (S1E and S1F Fig), in LIHC-TCGA and LIVER-TCGA datasets, analysed with SurvExpress database.

### Kidney cancer

Renal cell carcinoma (RCC) and transitional cell carcinoma of renal pelvis (TCC), also known as urothelial cell carcinoma, are two main subtypes of kidney cancer. Clear cell, papillary and chromophobe are the most common histological subtypes of RCC. The RCC is the major type of renal carcinoma which accounts for more than 80% of all renal cancer types whereas TCC is very rare and accounts for nearly 10% of the total renal cancer types.

#### IQGAP2 and IQGAP3 expression in kidney cancer

Low expression of IQGAP2 was observed in papillary RCC and clear RCC in different datasets of Oncomine. TCGA datasets from Xena browser also showed similar expression pattern of IQGAP2 in KIRP-TCGA and KIRC-TCGA datasets (S4 Table). The threshold level of IQGAP3 expression was not reached by any of the datasets available in Oncomine, but the TCGA datasets (KIRP-TCGA and KIRC-TCGA) available with Xena browser, showed high expression of IQGAP3 in kidney cancer, compared to the normal tissues.

The survival data for kidney cancer was analysed from KIPAN-TCGA and KIRC-TCGA datasets. Both the datasets showed low OS of the patients with higher expression of IQGAP3 (S1I and S1J Fig) and reduced expression of IQGAP2 (S1G and S1H Fig), as analysed with SurvExpress database.

### Expression of IQGAP2 and IQGAP3 in different stages of cancer

The mRNA expression of IQGAP2 and IQGAP3 was analysed among different cancer stages (I to IV) in lung, breast, stomach, colorectal, prostate, liver and kidney cancer, whereas the expression in brain cancer was compared between the histologic grades (G2 and G3) because of the lack of stage wise data. We observed a significant change in expression of IQGAP2 between stage I to II in breast cancer and liver cancer and, between stage II to III in breast, colorectal, prostate, liver and kidney cancer. In all the cancers the expression was lower in higher stage, except stage II to III of breast cancer. Distinct results were obtained in brain cancer where the expression was found to be significantly high in high grade (G3), compared to the low grade of cancer (G2). Besides this, in lung cancer the expression was not significantly altered between the stages (S2 Fig).

In opposite to IQGAP2, the expression of IQGAP3 was increased from stage I to II in lung and breast cancer and, stage II to III in prostate and kidney cancer. In brain cancer expression increased from G2 to G3. No significance was observed between the stages of stomach and liver cancer (S3 Fig). Based on this data we speculate that the role of IQGAP2 and IQGAP3 in initiation or progression might be tissue specific. IQGAP2 might be playing role in initiation in breast cancer, lung cancer and liver cancer whereas it promotes tumor progression in colorectal, brain and kidney cancer. Likewise, IQGAP3 might be playing role in initiation in lung, breast, stomach, colorectal and liver cancer whereas it supports progression of brain, prostate and kidney cancer.

### IQGAP2 and IQGAP3 mutations and copy number alterations

We selected TCGA datasets of all eight cancers to study mutation and copy number change in IQGAP2 and IQGAP3. A total of 10 studies were included for the analysis (S5). We observed different frequency of alterations in gene copy number, which included either deletion or amplification, and somatic mutation. The percentage of each type of IQGAP2 and IQGAP3 genetic alteration with cancer type has been summarised in S5 Table.

In IQGAP3, high gene amplification was observed in breast cancer (20.7% in Metabric and 11.9% in TCGA dataset), lung adenocarcinoma (12.6%) and liver cancer (12.3%), whereas in brain, lung squamous cell carcinoma, prostate, kidney and stomach cancer, the frequency of copy number change was very low. Furthermore, to see the effect of gene amplification on gene expression, in those cancers where the copy number was high, a correlation analysis was done, which showed positive association between the two (S4 Fig). Mutation analysis of IQGAP3 showed high frequency of mutations in colorectal (5.2%), lung squamous cell carcinoma (7.3%) and stomach cancer (5.2%), where the frequency of copy number change was very low. Here it is noteworthy that one frequent mutation (V293I/X293_splice) was present in four stomach cancer cases, at the splice site of IQGAP3.

On the other hand, IQGAP2 copy number alteration was not significantly changed in cancers, except prostate cancer where reduction of copy number was observed in 4.2% cases. Besides copy number change, the overall frequency of mutation was high in lung adenocarcinoma, lung squamous cell carcinoma and colorectal cancer, but in depth analysis did not show high frequency of any single mutation (S5 Fig).

### Methylation status of IQGAP2 and IQGAP3 promoters

The datasets used to analyse the methylation pattern at CpG islands of promotor region for IQGAP2 and IQGAP3 are summarised in S6 and S7 Tables. First, we compared the methylation pattern at promotor region of normal vs cancer specimen in lung, breast, liver, brain, prostate, kidney and colorectal cancer. All the probes showing significant differences between cancer and normal (summarized in S6 and S7 Tables), were further used for the correlation analyses with the mRNA expression levels. We observed that the methylation pattern was significantly altered in all the cancer types, except Glioblastoma multiforme and stomach cancer, which had the low normal sample size (n = 2). We observed a weak correlation between the methylation pattern and mRNA expression in all cancer types for IQGAP2 and IQGAP3 promoter region (S6, S7).

### IQGAP2 and IQGAP3 expression validation by IHC

**Fig 7:**
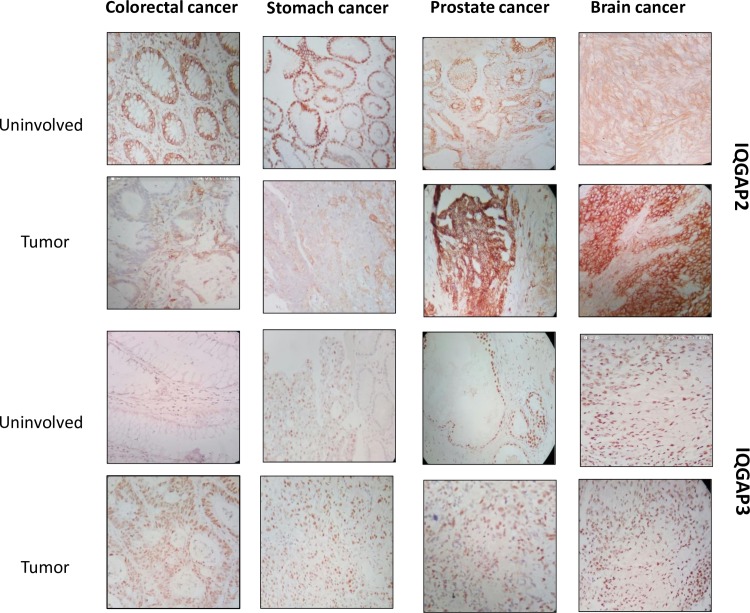
Representative images of immunohistochemistry for IQGAP2 and IQGAP3 expression analysis in cancers. These images are representative area of tumor or uninvolved staining. The brown color represents positive staining. IQGAP2 expression is predominant in cytoplasm and membrane where in IQGAP3 is abundantly present in nucleus. Expression of IQGAP2 is reduced in tumor tissue compared to uninvolved adjacent area in colorectal cancer and gastric cancer while in prostate and brain cancer the pattern is opposite. IQGAP3 expression is elevated in cancer tissue compared to the uninvolved area in all four types of cancer under investigation.

To validate the in-silico analysis results we ascertained the expression and localization of IQGAP2 and IQGAP3 in cancer patients, using immunohistochemistry. Expression was determined in tumor tissues with adjacent uninvolved tissues, in stomach, colorectal, prostate and brain cancer. We observed that IQGAP2 was predominantly localized in the cytoplasm and plasma membrane whereas; IQGAP3 was localized predominantly in the nucleus. Fig 7 (Fig 7) shows the representative images of IQGAP3 and IQGAP2 staining in tumor and uninvolved tissue.

**Fig 8:**
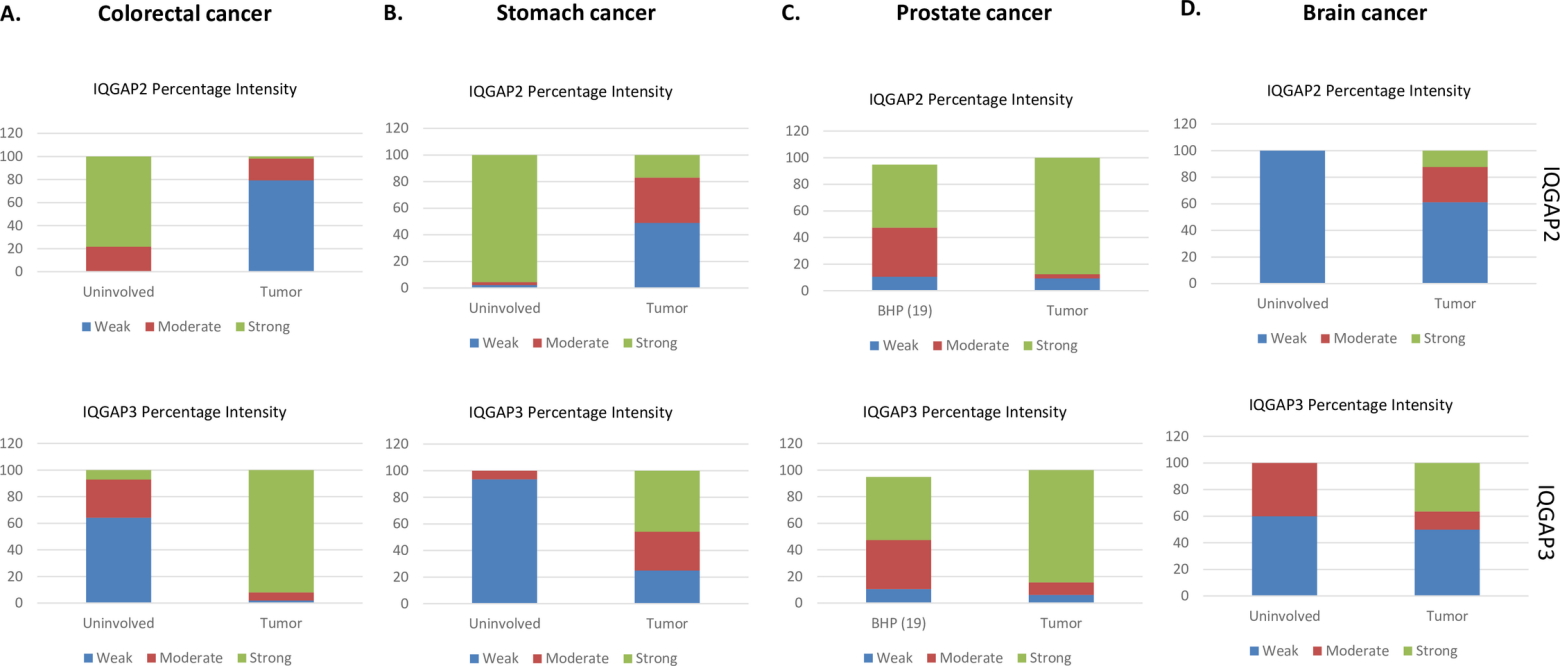
Comparison of IQGAP2 and IQGAP3 expression in normal vs cancer tissue using immunohistochemistry (IHC). Bar graphs show staining intensity of IQGAP2 or IQGAP3 in cancer samples and uninvolved area. Different color in bar represent the intensity of staining i.e. blue color- weak intensity, red color-moderate intensity and green color- strong intensity. X-axis represents the type of tissue i.e. uninvolved and tumor. Y-axis represents the percentage of patients positive for particular type of intensity. A, B, C and D showing expression pattern of IQGAP2 (upper panel) and IQGAP3 (lower panel) in normal vs tumor specimen in colorectal cancer, stomach cancer, prostate cancer and brain cancer, respectively.

The expression of IQGAP3 was found higher in stomach cancer (22/48), colorectal cancer (46/50), prostate cancer (27/32) and brain cancer (16/44) compared to the uninvolved tissue (Fig 8). In stomach cancer, IQGAP3 staining was strong in 45.8% and moderate in 29.2% cases. On the other hand, staining in uninvolved area was mostly weak, in 93.6% cases. In colorectal cancer, most of the cases (92%) showed strong staining for IQGAP3, compared to 7% in uninvolved tissue. Strong staining was present in 36.7% of brain tumor samples, whereas no uninvolved area showed strong staining for IQGAP3. As no uninvolved are was available for prostate cancer, we used BPH tissue as control. We observed that 84.4% prostate tissue had strong staining for IQGAP3 compared to 47.4% in BPH tissue.

In contrast to IQGAP3, IQGAP2 expression was low in colorectal (42/53) and stomach cancer (23/47) whereas the expression was high in prostate (28/32) and brain cancer (19/49) in tumor tissue compared to uninvolved tissue (Fig 8). In colorectal cancer only 1.9% tumor samples showed strong staining for IQGAP2, compared to 78.2% of uninvolved tissue. Similarly, 17% of stomach tumor cases showed strong staining for IQGAP2, in comparison to 95.56% of uninvolved tissue samples. On the contrary, 87.5% of prostate cancer sample showed strong staining for IQGAP2 than 47.37% of BPH samples. Likewise, in brain tumor 12.25%, 26.5% and, 61.2% samples showed strong, moderate, and weak staining for IQGAP2, respectively. On the other hand, all uninvolved tissue showed weak staining.

It is noteworthy that all above-mentioned results, from *in-vivo* analysis, are completely in accordance with the data obtained by *in-silico* analysis.

## Discussion

Scaffolding protein, IQGAPs play crucial role in cell proliferation, cell- cell adhesion, cell migration, exocytosis and endocytosis by regulating various cellular signalling pathways i.e. RTK signalling, β-arrestin and GPCR signalling, Wnt signalling etc [1].

In the present study, we have checked differences in mRNA levels of IQGAP2 and IQGAP3 between tumor and normal tissues in the most commonly occurring human cancers viz. lung, breast, gastric, colorectal, brain, prostate, liver and kidney cancers and, their subtypes, using Oncomine and TCGA databases. Additionally, the prognostic significance of two IQGAPs was also determined via Kaplan-Meier Plotter and SurvExpress database.

Among the different cancer types and their respective sub-types, we mostly found reduced and elevated mRNA levels of IQGAP2 and IQGAP3, respectively, which coincided with poor survival rates of patients. For instance, IQGAP2 was mostly downregulated in different cancer types viz. breast, lung, gastric, colorectal, liver and kidney. On the other hand, IQGAP3 was consistently up-regulated across most of the cancer types including breast, lung, gastric, colorectal, brain, prostate, liver and kidney. This expression pattern of these two IQGAP isoforms across different cancer types supports the notion that IQGAP2 possibly plays the role of a tumor suppressor gene whereas IQGAP3 is more likely to be an oncogene. These findings are further substantiated by existing literature [11–18].

This pattern was consistent regardless of subtypes across most of the cancer types, except breast cancer wherein IQGAP2 was specifically downregulated in ductal carcinoma and infiltrating ductal/ invasive ductal carcinoma but not in infiltrating lobular or mixed type breast carcinoma. Based on these observations, we speculated that IQGAP2 might play a crucial role in ER positive ductal breast carcinoma. It is noteworthy to mention that the most studied member of the IQGAP family viz. IQGAP1 has already been reported to interact with ER-Alpha and subsequently playing a role in ER mediated cellular processes [33]. Our finding suggests that IQGAP2 also merits future investigation with regards to its role in progression of breast tumors of ductal origin. This could shed further light on its prospects as a therapeutic target and potential biomarker for disease progression and prognosis.

Interestingly, in contrast to the expression pattern in other cancers, IQGAP2 mRNA levels were upregulated in all major subtypes of primary brain cancers. For IQGAP3, higher expression levels were reported in glioblastoma subtype only. As far as prognosis is concerned, increase in both IQGAP2 and IQGAP3 mRNA levels correlated with poor overall survival of brain cancer patients. It is noteworthy to mention that glioblastoma (GBM) represent a highly aggressive form of tumor. It would be interesting to prospect the opposing role of IQGAP2 in this particular cancer, wherein *in-vitro* studies could further help in identifying the role of IQGAP2 as a putative oncogene, along with IQGAP3. Similarly, the expression levels of IQGAP2 in prostate cancer were found to be upregulated in Oncomine. However, a recent study, albeit with limited number of prostate tumor samples, has reported reduction in IQGAP2 at both mRNA and protein level, in prostate cancer [12]. In this regard, future studies with higher sample size might help in providing a better perspective on the role of IQGAP2 in prostate cancer.

In agreement with our data-mining findings for lung cancer, Yang *et al. [15]* observed increased IQGAP3 expression in lung cancer tissues at both mRNA and protein levels. They showed that overexpression of IQGAP3 promoted tumor cell growth, migration and invasion. Similar studies were carried out in pancreatic cancer as well [18] wherein elevated levels of IQGAP3 in pancreatic cancer tissues correlated with increased proliferation, migration and invasion potential. Furthermore, the higher expression levels of IQGAP3 correlated with poor prognosis of pancreatic cancer patients. In agreement with our observations with *in-silico* analysis, a recent study [17] has reported upregulation of protein levels of IQGAP3 in breast cancer and its role as an oncogene.

Hence, experimental data from previous studies indicate that elevated IQGAP3 levels could play a protumorigenic role in multiple cancers and predict poor survival; supporting our datamining results.

Likewise for IQGAP2 previous studies have identified its role as a tumor suppressor in ovarian cancer [14] wherein reduced levels of IQGAP2 correlated with poor overall survival of patients and IQGAP2 inhibited EMT, migration and invasion. Moreover, in agreement with our analysis in liver cancer, *Xia et al*. [34] have previously established that reduced IQGAP2 levels lead to poor overall survival of HCC patients and it correlated with larger tumor size, advanced TNM stage and tumor differentiation. Likewise, a xenograft study undertaken by Xie *et al*. [12] with prostate cancer further substantiated the role of IQGAP2 as a tumor suppressor in mouse model.

Collectively, a careful summarisation of previously reported data and key indications for our *in-silico* investigation suggest that IQGAP2 might play a role as a tumor suppressor other cancer types as well.

Regarding the role of IQGAP2/ IQGAP3 in initiation of carcinogenesis, Although we found changes in expression of these two genes among stages, but any consistent trend could not be inferred. Data suggests that in cancer specific manner they may have role in initiation or progression. In support of our study, a single study has prospected the role of relative levels of IQGAP1/IQGAP3 in driving epidermal homeostasis towards neoplasia. In this study, the authors observed that diminished, sub-physiological levels of IQGAP1 and IQGAP3 were sufficient to maintain normal epidermal architecture. However, significantly higher levels of both isoforms are essential for the initiation and progression of invasive dermal neoplasia.IQGAP2 expression is absent in skin but predominant in liver and gastro-intestinal tract [7], hence one could speculate that IQGAP2 might play a tissue-specific role opposed to IQGAP1 in initiation of carcinogenesis in those tissues wherein the expression of both are reciprocally altered viz. HCC [13].

Overall, we observed that while IQGAP3 was elevated in most cancer types at both mRNA and protein level, IQGAP2 was reduced in expression. We could also strengthen our in-silico findings by its validation through *in-vivo* analysis using IHC in tumor and uninvolved tissue. To ascertain the cause of this observation, we looked for copy number alterations, methylation status and presence of somatic mutations in promoter region of both the genes. We observed that high frequency of mutations were present in IQGAP3 promoter but not in IQGAP2 promoter in different cancers. Interestingly, one frequent mutation (V293I/X293_splice) was present in four stomach cancer cases, at the splice site of IQGAP3. Furthermore, high gene amplification was observed for IQGAP3 in breast, lung and liver cancer, which further showed positive correlation with gene expression in the same. Moreover, IQGAP2 gene copy number was also altered in prostate cancer, which does not correlate with the observed high expression levels in our *in-silico* and *in-vivo* data. Previous report for prostate cancer also reports showed just opposite IQGAP2 expression levels, warranting further study [12]. For both IQGAP2 and IQGAP3, a weak correlation was observed between promoter methylation and subsequent gene expression in all cancer types. So, we can conclude that observed upregulation of IQGAP3 can be attributed, atleast partly, to gene amplification in some cancers. In other cancer types regulation of IQGAP3 levels can occur at transcriptional levels and beyond. The observed mutations in IQGAP3 promoter could well provide additional means via which IQGAP3 expression is regulated. Possibility of epigenetic regulation IQGAP3 expression, could not be established by our in-silico data, as we didn’t find any correlation between IQGAP3 expression and promoter methylation in cancers. Which indicates towards either absence of epigenetic regulation or presence of alternative mechanism which needs further investigation. For IQGAP2, no significant correlation of reduced expression with promoter methylation was observed. IQGAP2 methylation is significantly associated with loss of the IQGAP2 expression in the primary gastric cancer tissues as well as gastric cancer cell lines [11], thereby leading to tumor invasion and a poor prognosis. An inverse correlation between IQGAP2 DNA methylation and mRNA expression was observed in ovarian cancer as well [14]. However, reduced levels of IQGAP2 in HCC were found to be independent of hypermethylation of the Iqgap2 promoter [13]. To our knowledge, no association between epigenetic activation of IQGAP3 gene has been investigated in previous studies. Selective downregulation of IQGAP2 in cancers might be related to somatic mutations, role of methylation remains to be ascertained.

In conclusion, the findings from this study support the notion that reduced expression of IQGAP2 and higher expression of IQGAP3 promotes cancer in various tissues, with both isoforms playing opposite roles with regards to progression of the disease, which is evident from the correlation with survivability of the patients. Interestingly, why IQGAP2 shows a reverse expression pattern in certain cancers viz. brain and prostate cancer or exhibits selective downregulation in ductal carcinomas among breast cancer warrants further investigation. Future studies, with focus on prospecting protein expression and localization pattern of these isoforms in larger cohort of cancer patients with parallel molecular studies to investigate the detailed mechanism of these IQGAPs in tumor initiation and development, are the need of the hour. This will pave the way for using IQGAP2 and IQGAP3 as promising therapeutic targets and novel prognostic biomarkers for human carcinomas in the near future.

## Supporting information

S1 Fig SurvExpress database showing overall patient survival (OS) in prostate, liver and kidney cancer with IQGAP2 and IQGAP3 expression. 1A and 1B show Kaplan Meier plot of OS of prostate cancer patients with IQGAP2 and IQGAP3 expression, respectively. Here on the top, p-value and CI has been shown for selected data. Survival risk curves are shown for each group; low and high risk groups are shown in green and red colors respectively. The X-axis represents the time (days) of the study. The number of samples not presenting the event at the matching time has been shown in rows with corresponding color. Box plot of IQGAP2 and IQGAP3 gene along risk groups obtained in the analysis has shown in right side of each Kaplain Meier plot. Here, X-axis shows each gene and a p-value of the expression differences between risk groups. The p-value is obtained from a t-test for two risk groups. The Y-axis shows the expression levels. 1C and 1D show Kaplan Meier plot of OS of liver cancer patients with IQGAP2 expression. 1E and 1F show Kaplan Meier plot of OS of liver cancer patients with IQGAP3 expression. 1G and 1H show Kaplan Meier plot of OS of kidney cancer patients with IQGAP2 expression. 1I and 1J show Kaplan Meier plot of OS of Kidney cancer patients with IQGAP3 expression.
(TIF)

S2 Fig Stage wise expression analysis of IQGAP3 in different cancers. Graph shows the stage wise IQGAP3 mRNA expression data of TGCA datasets in different stages of cancer. Cancer type and its source TCGA dataset name are heighted above the box-plot. Here x-axis represents the log2 normalised mRNA expression of gene whereas Y-axis shows the pathological stage of cancer.
(TIF)

S3 Fig Stage wise expression analysis of IQGAP2 in different cancers. Graph shows stage wise mRNA expression data of TGCA in different stages of cancer. Cancer type and its source TCGA dataset name are heighted above the box-plot. Here X-axis represents the log2 normalised mRNA expression of gene whereas Y-axis shows the pathological stage of cancer.
(TIF)

S4 Fig Box and whisker plot showing the correlation between mRNA levels and copy number of IQGAP3. In the plot, X-axis represents putative copy number alterations and Y-axis shows the mRNA expression Z-scores. A. and B. show the correlation between mRNA and copy number in Breast cancer-TCGA, Cell 2015 dataset and Breast cancer-Metabric dataset, respectively, whereas C. and D. represent correlation in lung cancer-TCGA and Liver cancer-TCGA datasets, respectively. In all the studies the expression of IQGAP3 is positively associated with the amplification of gene.
(TIF)

S5 Fig Frequency of genetic alterations in IQGAP2 and IQGAP3 in different cancers. A. and B. show percentage frequency of IQGAP2 and IQGAP3 alteration in different cancer types, respectively. Different color represent genetic alteration type i.e. red–gene amplification, blue- deletion, green-mutation and grey-multiple alterations. Y-axis show the percentage of genetic alteration in each cancer type whereas x-axis represents the cancer studies selected for analysis. C. and D. show a graphical summary for all nonsynonymous mutations identified in IQGAP2 and IQGAP3, respectively. This graphical summary displays the position and frequency of all mutations in the context of Pfam protein domains encoded by the canonical gene isoform. In graphical view of IQGAP3 (C.), the most frequent mutation at 293_splice site has been shown in purple color.
(TIF)

S1 Table Kaplan-Meier plotter and SurvExpress showing the correlation between IQGAP2/IQGAP3 and survival outcomes in different cancer types. *Footnotes- Abbreviations used: KMP-Kaplan Meier Plotter, SurvExp-SurvExpress, (OS) Overall patient survival, (FP) First progression, (PPS) Post progression survival, (DMFS) Distance metastasis free survival, (RFS) Relapse free survival.
(DOCX)

S2 Table IQGAP2 and IQGAP3 mRNA expression in colorectal cancer. *Footnotes- TCGA Datasets (version: 2016-08-16) has been represented with (*) asterisk mark.
(DOCX)

S3 Table The mRNA expression analysis in Brain and CNS cancer. *Footnotes- TCGA Datasets (version: 2016-08-16) has been represented with (*) asterisk mark.
(DOCX)

S4 Table The mRNA expression of IQGAP2 and IQGAP3 in prostate, liver and kidney cancer. *Footnotes- TCGA Datasets (version: 2016-08-16) has been represented with (*) asterisk mark.
(DOCX)

S5 Table Genomic alterations associated with IQGAP2 and IQGAP3 in cancers. Abbreviations used: N = Total number of cases selected in a study, n = Number of cases showing genetic alterations.
(DOCX)

S6 Table Methylation status of IQGAP2 at promoter region and its correlation with the mRNA expression. (DOCX)

S7 Table Methylation status of IQGAP3 at promoter region and its correlation with the mRNA expression. (DOCX)
