# Supplementary figures and images for: Reduced expression of IQGAP2 and higher expression of IQGAP3 correlates with poor prognosis in cancers
Source: PLoS One. 2017 Oct 26;12(10):e0186977. doi: 10.1371/journal.pone.0186977 (PMC5658114; doi:10.1371/journal.pone.0186977)

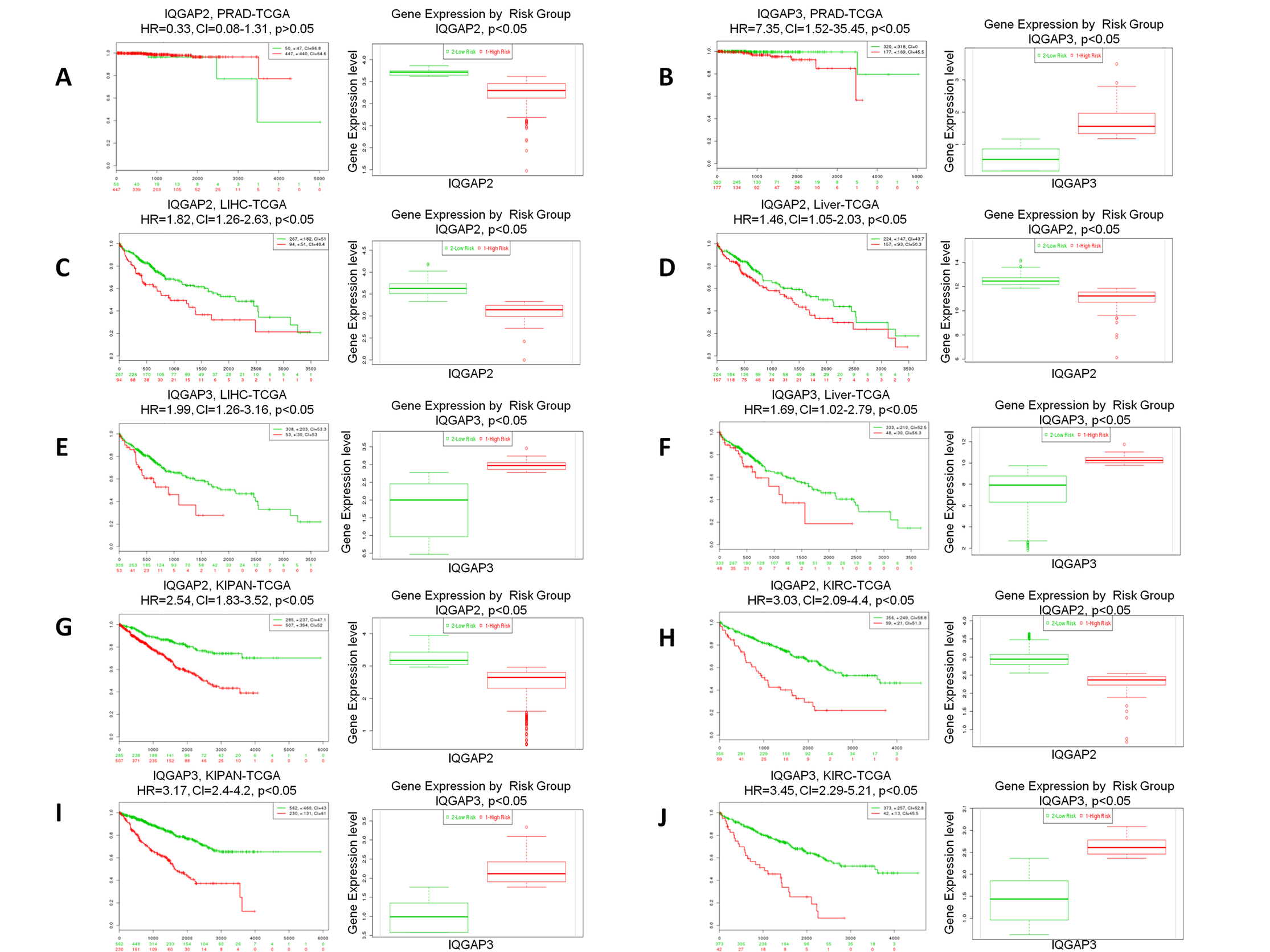

Supplement: S1 Fig — 1A and 1B show Kaplan Meier plot of OS of prostate cancer patients with IQGAP2 and IQGAP3 expression, respectively. Here on the top, p-value and CI has been shown for selected data. Survival risk curves are shown for each group; low and high risk groups are shown in green and red colors respectively. The X-axis represents the time (days) of the study. The number of samples not presenting the event at the matching time has been shown in rows with corresponding color. Box plot of IQGAP2 and IQGAP3 gene along risk groups obtained in the analysis has shown in right side of each Kaplain Meier plot. Here, X-axis shows each gene and a p-value of the expression differences between risk groups. The p-value is obtained from a t-test for two risk groups. The Y-axis shows the expression levels. 1C and 1D show Kaplan Meier plot of OS of liver cancer patients with IQGAP2 expression. 1E and 1F show Kaplan Meier plot of OS of liver cancer patients with IQGAP3 expression. 1G and 1H show Kaplan Meier plot of OS of kidney cancer patients with IQGAP2 expression. 1I and 1J show Kaplan Meier plot of OS of Kidney cancer patients with IQGAP3 expression. (TIF) [file pone.0186977.s001.tif]

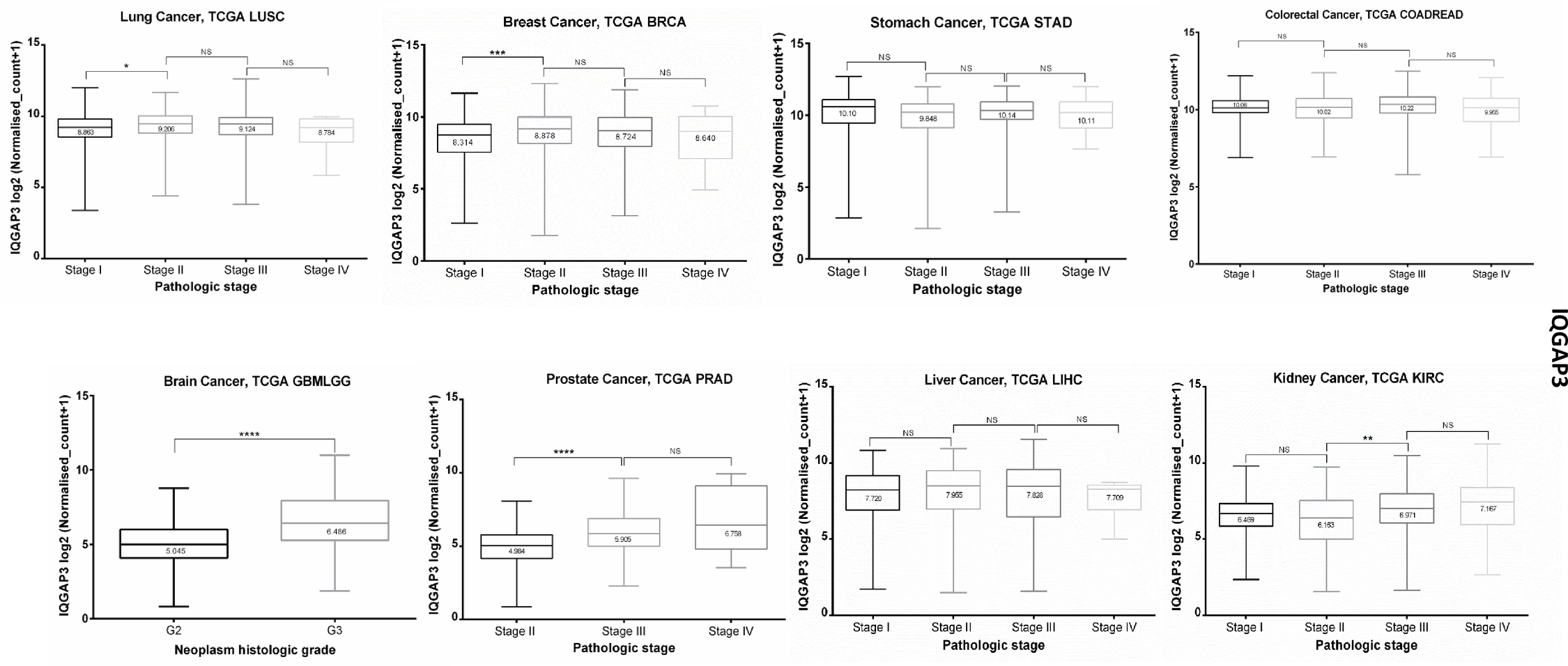

Supplement: S2 Fig — Graph shows the stage wise IQGAP3 mRNA expression data of TGCA datasets in different stages of cancer. Cancer type and its source TCGA dataset name are heighted above the box-plot. Here x-axis represents the log2 normalised mRNA expression of gene whereas Y-axis shows the pathological stage of cancer. (TIF) [file pone.0186977.s002.tif]

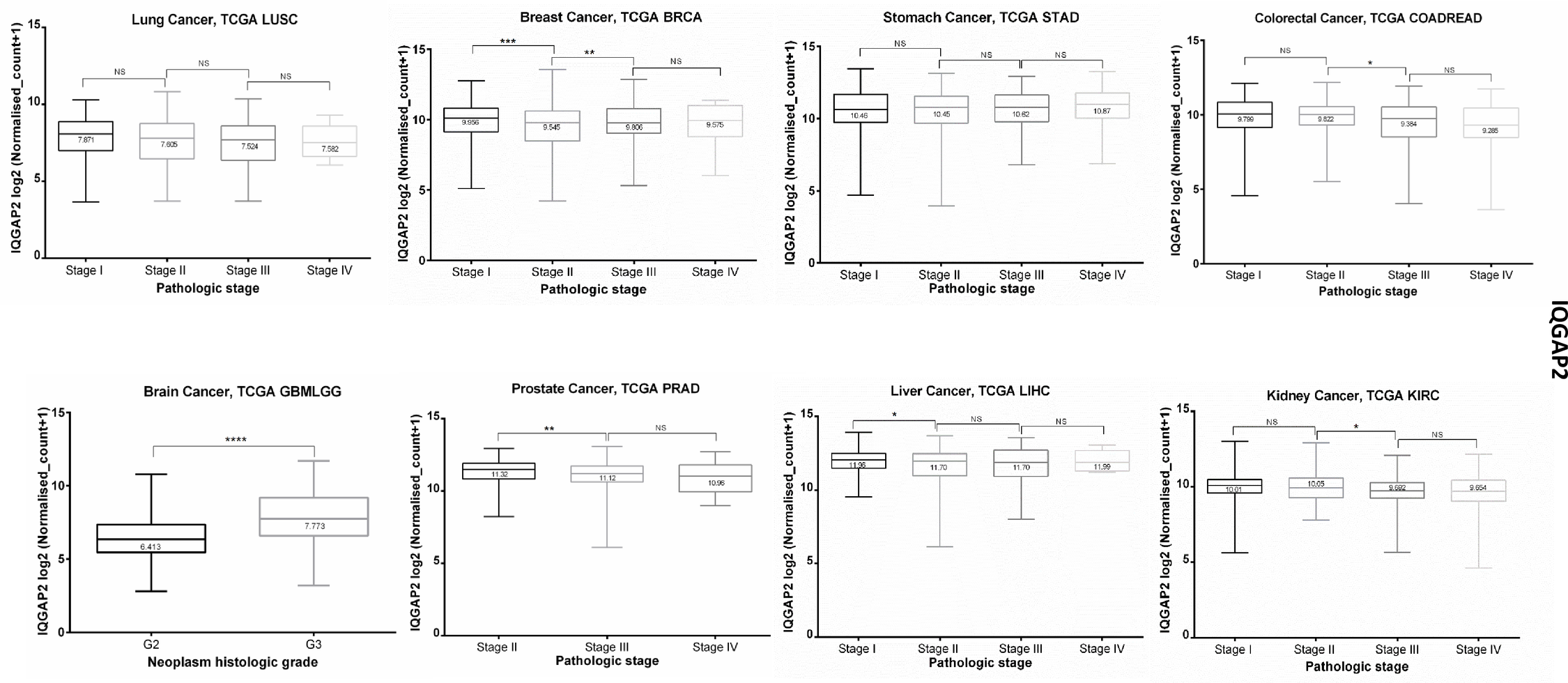

Supplement: S3 Fig — Graph shows stage wise mRNA expression data of TGCA in different stages of cancer. Cancer type and its source TCGA dataset name are heighted above the box-plot. Here X-axis represents the log2 normalised mRNA expression of gene whereas Y-axis shows the pathological stage of cancer. (TIF) [file pone.0186977.s003.tif]

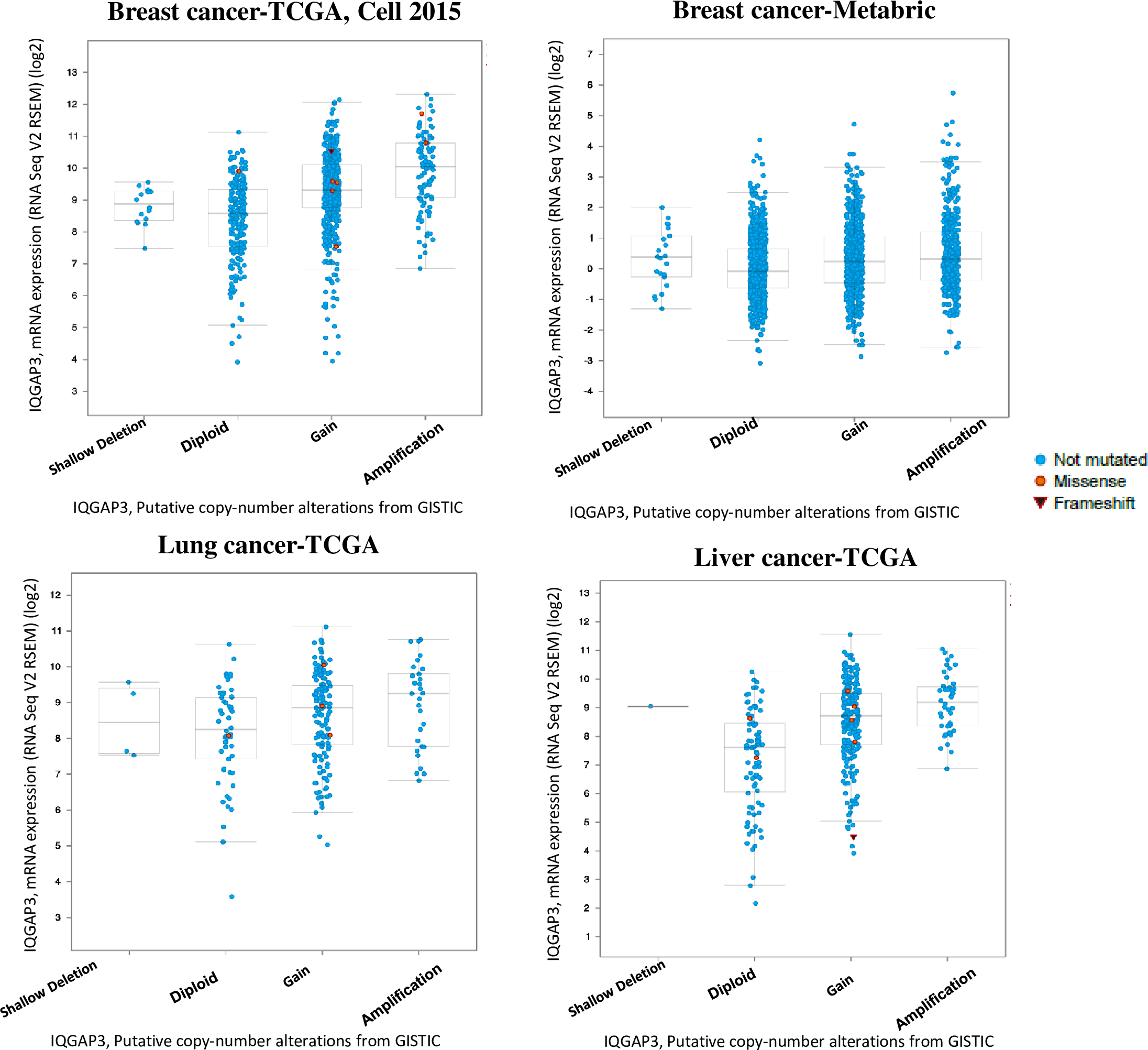

Supplement: S4 Fig — In the plot, X-axis represents putative copy number alterations and Y-axis shows the mRNA expression Z-scores. A. and B. show the correlation between mRNA and copy number in Breast cancer-TCGA, Cell 2015 dataset and Breast cancer-Metabric dataset, respectively, whereas C. and D. represent correlation in lung cancer-TCGA and Liver cancer-TCGA datasets, respectively. In all the studies the expression of IQGAP3 is positively associated with the amplification of gene. (TIF) [file pone.0186977.s004.tif]

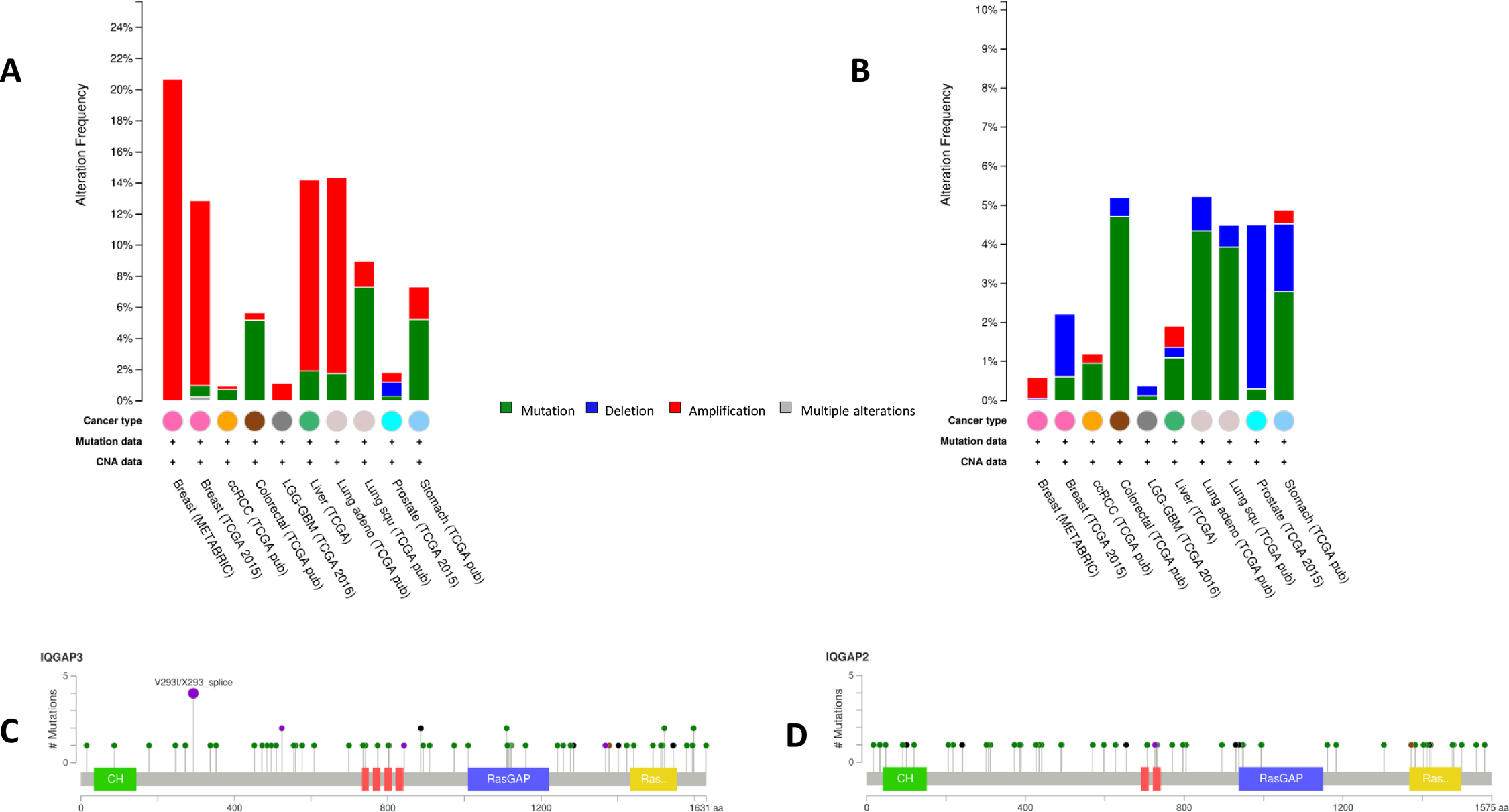

Supplement: S5 Fig — A. and B. show percentage frequency of IQGAP2 and IQGAP3 alteration in different cancer types, respectively. Different color represent genetic alteration type i.e. red–gene amplification, blue- deletion, green-mutation and grey-multiple alterations. Y-axis show the percentage of genetic alteration in each cancer type whereas x-axis represents the cancer studies selected for analysis. C. and D. show a graphical summary for all nonsynonymous mutations identified in IQGAP2 and IQGAP3, respectively. This graphical summary displays the position and frequency of all mutations in the context of Pfam protein domains encoded by the canonical gene isoform. In graphical view of IQGAP3 (C.), the most frequent mutation at 293_splice site has been shown in purple color. (TIF) [file pone.0186977.s005.tif]
