# Supplementary material for: Reduced expression of IQGAP2 and higher expression of IQGAP3 correlates with poor prognosis in cancers
Source: PLoS One. 2017 Oct 26;12(10):e0186977. doi: 10.1371/journal.pone.0186977 (PMC5658114; doi:10.1371/journal.pone.0186977)
Supplement: S1 Table — *Footnotes- Abbreviations used: KMP-Kaplan Meier Plotter, SurvExp-SurvExpress, (OS) Overall patient survival, (FP) First progression, (PPS) Post progression survival, (DMFS) Distance metastasis free survival, (RFS) Relapse free survival. (DOCX) [file pone.0186977.s006.docx]

| **Cancer type** | **Gene** | **Database** | **Dataset/**  **Affymetrix ID** | **Survival outcome** | **No. of Cases** | **HR** | **95% CI** | ***p*-value** |
| --- | --- | --- | --- | --- | --- | --- | --- | --- |
| Breast | IQGAP2 | KMP | 203474_at | RFS | 3951 | 0.78 | 0.69-0.86 | 4.5e-06 |
|  |  |  |  | OS | 1402 | 0.72 | 0.58-0.9 | 0.0029 |
|  |  |  |  | DMFS | 1746 | 0.70 | 0.57-0.85 | 0.0002 |
|  |  |  |  | PPS | 414 | 0.87 | 0.68-1.1 | 0.24 |
|  | IQGAP3 | KMP | 229538_s_at | RFS | 1764 | 1.53 | 1.31-1.79 | 6.3e-08 |
|  |  |  |  | OS | 626 | 1.57 | 1.14-2.15 | 0.0051 |
|  |  |  |  | DMFS | 664 | 1.44 | 1.04-2.0 | 0.026 |
|  |  |  |  | PPS | 173 | 1.27 | 0.89-1.81 | 0.18 |
| Lung | IQGAP2 | KMP | 203474_at | OS | 1926 | 0.71 | 0.63-0.81 | 1.2e-07 |
|  |  |  |  | FP | 982 | 0.82 | 0.68-0.99 | 0.04 |
|  |  |  |  | PPS | 344 | 0.68 | 0.53-0.87 | 0.0026 |
|  | IQGAP3 | KMP | 229538_s_at | OS | 1145 | 1.58 | 1.34-1.87 | 4.4e-08 |
|  |  |  |  | FP | 596 | 1.99 | 1.51-2.63 | 6.7e-07 |
|  |  |  |  | PPS | 138 | 1.41 | 0.92-2.18 | 0.12 |
| Gastric | IQGAP2 | KMP | 203474_at | OS | 876 | 0.58 | 0.48-0.72 | 1.6e-07 |
|  |  |  |  | FP | 641 | 0.59 | 0.47-0.74 | 3.4e-06 |
|  | IQGAP3 | KMP | 229538_s_at | OS | 631 | 0.83 | 0.65-1.05 | 0.12 |
|  |  |  |  | FP | 522 | 0.73 | 0.57-0.93 | 0.009 |
|  |  | KMP | 1569061_at | OS | 631 | 1.54 | 1.24-1.93 | 0.00012 |
|  |  |  |  | FP | 641 | 1.33 | 1.03-1.72 | 0.031 |
| Colorectal | IQGAP2 | SurvExp | COAD-TCGA | OS | 351 | 1.49 | 0.83-2.67 | 0.181 |
|  |  | SurvExp | COADREAD-TCGA | OS | 467 | 1.32 | 0.84-2.07 | 0.23 |
|  | IQGAP3 | SurvExp | COAD-TCGA | OS | 351 | 1.54 | 0.96-2.47 | 0.068 |
|  |  | SurvExp | COADREAD-TCGA | OS | 467 | 1.55 | 0.97-2.48 | 0.068 |
| Brain | IQGAP2 | SurvExp | LGG-TCGA | OS | 512 | 4.58 | 3.16-6.65 | 1.2e-15 |
|  |  | SurvExp | GBMLGG-TCGA | OS | 660 | 5.65 | 3.61-8.85 | 3.7e-14 |
|  | IQGAP3 | SurvExp | LGG-TCGA | OS | 512 | 5.08 | 3.42-7.55 | 7.7e-16 |
|  |  | SurvExp | GBMLGG-TCGA | OS | 660 | 5.49 | 3.51-8.59 | 9.3e-14 |
| Prostate | IQGAP2 | SurvExp | PRAD-TCGA | OS | 497 | 0.33 | 0.08-1.31 | 0.114 |
|  | IQGAP3 | SurvExp | PRAD-TCGA | OS | 497 | 7.35 | 1.52-35.4 | 0.013 |
| Liver | IQGAP2 | SurvExp | LIHC-TCGA | OS | 361 | 1.82 | 1.26-2.63 | 0.001 |
|  |  | SurvExp | TCGA-LIVER | OS | 422 | 1.46 | 1.05-2.03 | 0.026 |
|  | IQGAP3 | SurvExp | LIHC-TCGA | OS | 361 | 1.99 | 1.26-3.16 | 0.001 |
|  |  | SurvExp | TCGA-LIVER | OS | 422 | 1.69 | 1.02-2.79 | 0.041 |
| Kidney | IQGAP2 | SurvExp | KIPAN-TCGA | OS | 792 | 2.54 | 1.83-3.52 | 2.4e-08 |
|  |  | SurvExp | KIRC-TCGA | OS | 415 | 3.03 | 2.09-4.4 | 5.9e-09 |
|  | IQGAP3 | SurvExp | KIPAN-TCGA | OS | 792 | 3.17 | 2.4-4.2 | 6.6e-16 |
|  |  | SurvExp | KIRC-TCGA | OS | 415 | 3.45 | 2.29-5.21 | 3.8e-09 |

**Supplementary Table S1: Kaplan-Meier plotter and SurvExpress showing the correlation between IQGAP2/IQGAP3 and survival outcomes in different cancer types**

**Abbreviations used:** KMP-Kaplan Meier Plotter, SurvExp-SurvExpress, (OS) Overall patient survival, (FP) First progression, (PPS) Post progression survival, (DMFS) Distance metastasis free survival, (RFS) Relapse free survival.
