# Supplementary material for: Reduced expression of IQGAP2 and higher expression of IQGAP3 correlates with poor prognosis in cancers
Source: PLoS One. 2017 Oct 26;12(10):e0186977. doi: 10.1371/journal.pone.0186977 (PMC5658114; doi:10.1371/journal.pone.0186977)
Supplement: S2 Table — *Footnotes- TCGA Datasets (version: 2016-08-16) has been represented with (*) asterisk mark. (DOCX) [file pone.0186977.s007.docx]

| **Gene** | **Dataset** | **Normal (Cases)** | **Tumor (Cases)** | **Fold change** | ***t*-Test** | ***p*-value** | ***Rank (Top 10%)*** |
| --- | --- | --- | --- | --- | --- | --- | --- |
| IQGAP2 | Kaiser Colon | Colon (5) | Colon Adenocarcinoma (41) | -3.25 | -12.60 | 2.2e-16 | 1% |
|  |  | Colon (5) | Cecum Adenocarcinoma (17) | -2.65 | -7.82 | 1.5e-7 | 2% |
|  |  | Colon (5) | Colon Mucinous Adenocarcinoma (13) | -2.13 | -8.08 | 4.1e-7 | 2% |
|  |  | Colon (5) | Rectal Mucinous Adenocarcinoma (4) | -2.86 | -10.28 | 3.6e-4 | 4% |
|  |  | Colon (5) | Rectosigmoid Adenocarcinoma (10) | -2.93 | -6.72 | 2.8e-5 | 4% |
|  |  | Colon (5) | Rectal Adenocarcinoma (8) | -2.66 | -4.73 | 9.2e-4 | 10% |
|  | Ki Colon | Colon (28)  Liver (13) | Colon Adenocarcinoma (50) | -2.82 | -8.99 | 2.1e-14 | 1% |
|  | Hong Colorectal | Colon (12) | Colorectal Carcinoma (70) | -5.39 | -14.57 | 7.1e-23 | 1% |
|  | Gaedcke Colorectal | Rectum (65) | Rectal Adenocarcinoma (65) | -3.87 | -19.88 | 1.2e-36 | 1% |
|  | TCGA Colorectal | Colon (19)  Rectum (3) | Colon Adenocarcinoma (101) | -3.52 | -14.52 | 1.1e-24 | 1% |
|  |  | Colon (19)  Rectum (3) | Rectosigmoid Adenocarcinoma (3) | -5.54 | -16.82 | 2.4e-9 | 2% |
|  |  | Colon (19)  Rectum (3) | Rectal Adenocarcinoma (60) | -3.58 | -11.94 | 1.0e-19 | 3% |
|  | Skrzypczak Colorectal 2 | Colon (10) | Colon Adenoma Epithelia (5) | -4.41 | -22.66 | 1.4e-7 | 2% |
|  |  | Colon (10) | Colon Carcinoma (5) | -2.73 | -12.18 | 5.4e-8 | 3% |
|  |  | Colon (10) | Colon Carcinoma Epithelia (5) | -2.19 | -15.69 | 5.2e-8 | 3% |
|  | TCGA-COADREAD* | Normal (39) | Colon Adenocarcinoma (243) | -2.04 | 16.20 | <0.0001 |  |
|  |  | Normal (2) | Colon mucinous Adenocarcinoma (37) | -1.18 | 9.60 | <0.0001 |  |
|  |  | Normal (8) | Rectal Adenocarcinoma (86) | -1.90 | 7.75 | <0.0001 |  |
|  |  | Normal (1) | Rectal mucinous Adenocarcinoma (5) | ND | ND | ND |  |
| IQGAP3 | TCGA Colorectal | Colon (19)  Rectum (3) | Rectal Mucinous Adenocarcinoma (6) | 2.38 | 6.86 | 7.7e-6 | 3% |
|  | Hong Colorectal | Colon (12) | Colorectal Carcinoma (70) | 2.58 | 5.83 | 1.7e-7 | 10% |
|  | TCGA-COADREAD* | Normal (39) | Colon Adenocarcinoma (243) | 2.03 | 14.72 | <0.0001 |  |
|  |  | Normal (2) | Colon mucinous Adenocarcinoma (37) | 2.01 | 1.78 | 0.32 |  |
|  |  | Normal (8) | Rectal Adenocarcinoma (86) | 2.25 | 6.40 | 0.002 |  |
|  |  | Normal (1) | Rectal mucinous Adenocarcinoma (5) | ND | ND | ND |  |
