# Supplementary material for: Reduced expression of IQGAP2 and higher expression of IQGAP3 correlates with poor prognosis in cancers
Source: PLoS One. 2017 Oct 26;12(10):e0186977. doi: 10.1371/journal.pone.0186977 (PMC5658114; doi:10.1371/journal.pone.0186977)
Supplement: S3 Table — *Footnotes- TCGA Datasets (version: 2016-08-16) has been represented with (*) asterisk mark. (DOCX) [file pone.0186977.s008.docx]

| **Gene** | **Dataset** | **Normal (Cases)** | **Tumor (Cases)** | **Fold change** | ***t*-Test** | ***p*-value** | ***Rank (Top 10%)*** |
| --- | --- | --- | --- | --- | --- | --- | --- |
| IQGAP2 | Sun Brain | Brain (23) | Glioblastoma (81) | 5.614 | 14.20 | 8.8e-24 | 1% |
|  |  | Brain (23) | Diffuse Astrocytoma (7) | 3.789 | 4.11 | 0.002 | 7% |
|  |  | Brain (23) | Anaplastic Astrocytoma (19) | 3.11 | 4.93 | 2.7e-5 | 8% |
|  |  | Brain (23) | Oligodendroglioma (50) | 2.05 | 4.79 | 4.3e-6 | 9% |
|  | French Brain | Brain (6) | Anaplastic Oligodendroglioma (23) | 2.77 | 6.29 | 7.5e-7 | 3% |
|  |  | Brain (6) | Anaplastic Oligoastrocytoma (4) | 4.83 | 7.12 | 8.3e-4 | 4% |
|  | Murat Brain | Brain (4) | Glioblastoma (80) | 4.36 | 13.85 | 1.3e-6 | 5% |
|  | TCGA Brain | Brain (10) | Glioblastoma (5) | 3.59 | 3.94 | 0.005 | 9% |
|  |  | Brain (10) | Brain Glioblastoma (542) | 4.78 | 12.99 | 7.5e-8 | 10% |
|  | TCGA-LGG* | Normal (5) | Glioblastoma (151) | 4.80 | 16.50 | <0.0001 |  |
|  |  | Normal (5) | Astrocytoma (194) | 3.55 | 11.55 | <0.0001 |  |
|  |  | Normal (5) | Oligodendroglioma (191) | 2.54 | 8.33 | 0.0002 |  |
|  |  | Normal (5) | Oligoastrocytoma (130) | 2.80 | 8.90 | <0.0001 |  |
| IQGAP3 | TCGA-LGG* | Normal (5) | Glioblastoma (151) | 3.08 | 6.34 | 0.0027 |  |
|  |  | Normal (5) | Astrocytoma (194) | 1.30 | 2.61 | 0.05 |  |
|  |  | Normal (5) | Oligodendroglioma (191) | 0.92 | 1.86 | 0.12 |  |
|  |  | Normal (5) | Oligoastrocytoma (130) | 1.09 | 2.17 | 0.08 |  |

**Supplementary Table S3: mRNA expression analysis in Brain and CNS cancer**

TCGA Datasets (version: 2016-08-16) has been represented with (*) asterisk mark
