# Supplementary material for: Reduced expression of IQGAP2 and higher expression of IQGAP3 correlates with poor prognosis in cancers
Source: PLoS One. 2017 Oct 26;12(10):e0186977. doi: 10.1371/journal.pone.0186977 (PMC5658114; doi:10.1371/journal.pone.0186977)
Supplement: S4 Table — *Footnotes- TCGA Datasets (version: 2016-08-16) has been represented with (*) asterisk mark. (DOCX) [file pone.0186977.s009.docx]

| **Gene** | **Dataset** | **Normal (Cases)** | **Tumor (Cases)** | **Fold change** | ***t*-Test** | ***p*-value** |
| --- | --- | --- | --- | --- | --- | --- |
| **Prostate Cancer** | | | | | | |
| IQGAP2 | Varambally Prostate | Prostate Gland (6) | Prostate Ca. (7) | 2.901 | 9.38 | 2.8e-6 |
|  | Welsh Prostate | Prostate Gland (9) | Prostate Carcinoma (25) | 2.064 | 7.83 | 1.7e-6 |
|  | Singh Prostate | Prostate Gland (50) | Prostate Ca. (52) | 2.716 | 3.79 | 1.3e-4 |
|  | Grasso Prostate | Prostate Gland (28) | Prostate Ca. (59) | 2.052 | 4.60 | 2.4e-5 |
|  | TCGA Prostate cancer (PRAD)***** | Prostate Gland (51) | Prostate Ca. acinar type (482) | 0.23 | 1.75 | 0.843 |
| IQGAP3 | TCGA Prostate cancer (PRAD)***** | Prostate Gland (51) | Prostate Ca. acinar type (482) | 1.937 | 10.42 | <0.00001 |
| **Liver Cancer** | | | | | | |
| IQGAP2 | TCGA Liver Cancer (LIHC)***** | Liver (49) | HCC (361) | -1.025 | 13.19 | <0.0001 |
| IQGAP3 | TCGA Liver Cancer (LIHC)***** | liver (49) | HCC (361) | 4.192 | 16.16 | <0.0001 |
| **Kidney Cancer** | | | | | | |
| IQGAP2 | Yesenko Renal | Fetal Kidney (2),  Kidney (3) | Papillary Renal Cell Ca. (19) | -3.568 | -7.448 | 1.1e-7 |
|  | Higgins Renal | Kidney (3) | Clear Renal Cell Ca. (25) | -2.105 | -7.750 | 2.4e-4 |
|  | Beroukhim Renal | Renal Cortex (10),  Renal Tissue (1) | Non-Hereditary Clear Renal Cell Ca. (27) | -2.886 | -6.690 | 1.6e-6 |
|  | Gumz Renal | Kidney (10) | Clear Renal Cell Ca. (10) | -2.598 | -5.359 | 2.7e-5 |
|  | Lenburg Renal | Kidney (9) | Clear Renal Cell Ca. (9) | -2.623 | -4.163 | 4.7e-4 |
|  | TCGA Kidney Papillary Cell Ca. (KIRP)***** | Kidney (32) | Papillary Renal Cell Ca. (290) | -2.225 | 9.877 | <0.0001 |
|  | TCGA Kidney Clear Cell Ca. (KIRC)***** | Kidney (72) | Clear Renal Cell Ca. (533) | -1.057 | 7.556 | <0.0001 |
| IQGAP3 | TCGA Kidney Papillary Cell Ca. (KIRP)***** | Kidney (32) | Papillary Renal Cell Ca. (290) | 2.67 | 13.57 | <0.0001 |
|  | TCGA Kidney Clear Cell Ca. (KIRC)***** | Kidney (72) | Clear Renal Cell Ca. (533) | 2.935 | 17.61 | <0.0001 |

**Supplementary Table S4: mRNA expression of IQGAP2 and IQGAP3 in Prostate, Liver and Kidney** **Cancer**.

TCGA Datasets (version: 2016-08-16) has been represented with (*) asterisk mark
