# Supplementary material for: Reduced expression of IQGAP2 and higher expression of IQGAP3 correlates with poor prognosis in cancers
Source: PLoS One. 2017 Oct 26;12(10):e0186977. doi: 10.1371/journal.pone.0186977 (PMC5658114; doi:10.1371/journal.pone.0186977)
Supplement: S5 Table — Abbreviations used: N = Total number of cases selected in a study, n = Number of cases showing genetic alterations. (DOCX) [file pone.0186977.s010.docx]

**Supplementary Table S5: Genomic alterations associated with IQGAP2 and IQGAP3 in cancers**

1. **Frequency of genomic alterations of IQGAP3 in cancers**

| **Data source** | **Cancer type** | **Number of cases**  **(N)** | **Alteration frequency (%) (n)** | **Amp (%) (n)** | **Del (%) (n)** | **Missense and other mutations (%)(n)** |
| --- | --- | --- | --- | --- | --- | --- |
| Metabric, Nature | Breast | 2051 | 20.7 (424) | **20.7** (424) | 0 | 0 |
| TCGA, Cell 2015 | Breast | 816 | 12.9 (105) | **11.9** (97) | 0 | 0.9 (8) |
| TCGA, Nature 2013 | Kidney | 418 | 1 (4) | 0.2 (1) | 0 | 0.7 (3) |
| TCGA, Nature 2012 | colorectal | 212 | 5.7 (12) | 0.5 (1) | 0 | **5.2** (11) |
| TCGA, Cell 2016 | Brain | 794 | 1.1 (9) | 1.1 (9) | 0 | 0 |
| TCGA Provisional | Liver | 366 | 14.2 (52) | **12.3** (45) | 0 | 1.9 (7) |
| TCGA, Nature 2014 | Lung | 230 | 14.3 (33) | **12.6** (29) | 0 | 1.7 (4) |
| TCGA, Nature 2012 | Lung | 178 | 9 (16) | 1.7 (3) | 0 | **7.3** (13) |
| TCGA, Cell 2015 | Prostate | 333 | 1.8 (6) | 0.6 (2) | 0.9 (3) | 0.3 (1) |
| TCGA, Nature 2014 | Stomach | 287 | 7.3 (21) | 2.1 (6) | 0 | **5.2** (15) |

1. **Frequency of genomic alterations of IQGAP2 in cancers**

| **Data source** | **Cancer type** | **Number of cases**  **(N)** | **Alteration frequency (%) (n)** | **Amp (%) (n)** | **Del (%) (n)** | **Missense and other mutations (%)(n)** |
| --- | --- | --- | --- | --- | --- | --- |
| Metabric, Nature | Breast | 2051 | 0.6 (12) | 0.5 (11) | 0 (1) | 0 |
| TCGA, Cell 2015 | Breast | 816 | 2.2 (18) | 0 | 1.6 (13) | 0.6 (5) |
| TCGA, Nature 2013 | Kidney | 418 | 1.2 (5) | 0.2 (1) | 0 | 1 (4) |
| TCGA, Nature 2012 | colorectal | 212 | 5.2 (11) | 0 | 0.5 (1) | **4.7** (10) |
| TCGA, Cell 2016 | Brain | 794 | 0.4 (3) | 0 | 0.3 (2) | 0.1 (1) |
| TCGA Provisional | Liver | 366 | 1.9 (7) | 0.5 (2) | 0.3 (1) | 1.1 (4) |
| TCGA, Nature 2014 | Lung | 230 | 5.2 (12) | 0 | 0.9 (2) | **4.3** (10) |
| TCGA, Nature 2012 | Lung | 178 | 4.5 (8) | 0 | 0.6 (1) | **3.9** (7) |
| TCGA, Cell 2015 | Prostate | 333 | 4.5 (15) | 0 | **4.2** (14) | 0.3 (1) |
| TCGA, Nature 2014 | Stomach | 287 | 4.9 (14) | 0.3 (1) | 1.7 (5) | - 1. (8) |

**Abbreviations used:** N=total number of cases selected in a study, n=Number of cases showing genetic alterations.
