# Supplementary material for: Reduced expression of IQGAP2 and higher expression of IQGAP3 correlates with poor prognosis in cancers
Source: PLoS One. 2017 Oct 26;12(10):e0186977. doi: 10.1371/journal.pone.0186977 (PMC5658114; doi:10.1371/journal.pone.0186977)
Supplement: S6 Table — (DOCX) [file pone.0186977.s011.docx]

**Supplementary Table S6: Methylation status of IQGAP2 at promoter region and its correlation with the mRNA expression**

| **Data source** | **Cancer type** | **Number of cases**  **(N)** | | **Probes significantly altered in methylation level at p≤ 0.05** | **Pearson coefficient (Meth vs mRNA)** | | **Correlation**  **(Strong/Weak)** |
| --- | --- | --- | --- | --- | --- | --- | --- |
|  |  | Normal | Cancer |  | Normal | Cancer |  |
| TCGA LUAD | Lung adenocarcinoma | 32 | 463 | cg19828169 | -0.097 | -0.21 | Weak |
|  |  |  |  | cg05060672 | 0.188 | -0.235 | Weak |
| TCGA LUSC | Lung squamous cell carcinoma | 43 | 361 | cg02294176 | 0.101 | 0.008 | Weak |
|  |  |  |  | cg19828169 | -0.09 | 0.029 | Weak |
|  |  |  |  | cg05060672 | 0.19 | -0.051 | Weak |
|  |  |  |  | cg02387679 | -0.248 | -0.182 | Weak |
| TCGA BRCA | Breast Invasive Carcinoma | 98 | 743 | cg19828169 | -0.049 | -0.202 | Weak |
|  |  |  |  | cg05060672 | 0.063 | -0.29 | Weak |
|  |  |  |  | cg02387679 | 0.119 | -0.359 | Weak |
| TCGA COAD | Colorectal Cancer | 38 | 302 | cg19828169 | 0.431 | -0.121 | Weak |
| TCGA STAD | Stomach Cancer | 2 | 339 | ns | - | - | - |
| TCGA KRIC | Kidney renal clear cell carcinoma | 160 | 324 | cg02294176 | -0.249 | -0.261 | Weak |
|  |  |  |  | cg19828169 | -0.08 | -0.158 | Weak |
|  |  |  |  | cg05060672 | -0.178 | -0.162 | Weak |
|  |  |  |  | cg02387679 | -0.232 | -0.4 | Weak |
| TCGA LIHC | Liver hepatocellular  carcinoma | 50 | 256 | cg26024851 | 0.458 | -0.004 | Weak |
|  |  |  |  | cg12262564 | 0.504 | 0.048 | Weak |
|  |  |  |  | cg12441221 | -0.173 | -0.147 | Weak |
|  |  |  |  | cg17722719 | -0.11 | -0.112 | Weak |
|  |  |  |  | cg12124478 | 0.437 | 0.054 | Weak |
| TCGA GBM | Glioblastoma multiforme | 2 | 129 | ns | - | - | - |
| TCGA PRAD | Prostate adenocarcinoma | 49 | 340 | cg02294176 | -0.249 | 0.116 | Weak |
|  |  |  |  | cg19828169 | -0.073 | 0.051 | Weak |
|  |  |  |  | cg05060672 | -0.408 | -0.067 | Weak |
|  |  |  |  | cg02387679 | -0.231 | -0.061 | Weak |
