# Supplementary material for: Reduced expression of IQGAP2 and higher expression of IQGAP3 correlates with poor prognosis in cancers
Source: PLoS One. 2017 Oct 26;12(10):e0186977. doi: 10.1371/journal.pone.0186977 (PMC5658114; doi:10.1371/journal.pone.0186977)
Supplement: S7 Table — (DOCX) [file pone.0186977.s012.docx]

| **Data source** | **Cancer type** | **Number of cases**  **(N)** | | **Probes significantly altered in methylation level at p≤ 0.05** | **Pearson coefficient (Meth vs mRNA)** | | **Correlation**  **(Strong/Weak)** |
| --- | --- | --- | --- | --- | --- | --- | --- |
|  |  | Normal | Cancer |  | Normal | Cancer |  |
| TCGA LUAD | Lung adenocarcinoma | 32 | 463 | cg26024851 | -0.106 | 0.019 | Weak |
|  |  |  |  | cg12441221 | 0.165 | -0.115 | Weak |
|  |  |  |  | cg17722719 | 0.084 | -0.01 | Weak |
| TCGA LUSC | Lung squamous cell carcinoma | 43 | 361 | cg26024851 | 0.124 | -0.136 | Weak |
|  |  |  |  | cg12441221 | 0.299 | -0.03 | Weak |
|  |  |  |  | cg17722719 | -0.195 | -0.117 | Weak |
|  |  |  |  | cg12124478 | 0.597 | -0.093 | Weak |
| TCGA BRCA | Breast Invasive Carcinoma | 98 | 743 | cg26024851 | -0.027 | 0.006 | Weak |
|  |  |  |  | cg12262564 | 0.002 | 0.008 | Weak |
|  |  |  |  | cg12441221 | -0.047 | -0.183 | Weak |
|  |  |  |  | cg17722719 | -0.113 | -0.1 | Weak |
| TCGA COAD | Colorectal Cancer | 38 | 302 | cg26024851 | 0.215 | -0.057 | Weak |
|  |  |  |  | cg12262564 | 0.228 | -0.092 | Weak |
| TCGA STAD | Stomach Cancer | 2 | 339 | ns | _ | _ | _ |
| TCGA KRIC | Kidney renal clear cell carc. | 160 | 324 | cg26024851 | 0.56 | 0.206 | Weak |
|  |  |  |  | cg12262564 | 0.67 | 0.222 | Weak |
|  |  |  |  | cg12441221 | -0.076 | -0.087 | Weak |
|  |  |  |  | cg17722719 | -0.209 | -0.094 | Weak |
|  |  |  |  | cg12124478 | 0.265 | 0.146 | Weak |
| TCGA LIHC | Liver hep. carcinoma | 50 | 256 | cg26024851 | 0.458 | -0.004 | Weak |
|  |  |  |  | cg12262564 | 0.504 | -0.048 | Weak |
|  |  |  |  | cg12441221 | -0.173 | -0.147 | Weak |
|  |  |  |  | cg17722719 | -0.11 | -0.112 | Weak |
|  |  |  |  | cg12124478 | 0.437 | 0.054 | Weak |
| TCGA GBM | Glioblastoma multiforme | 2 | 129 | ns | _ | _ | _ |
| TCGA PRAD | Prostate adenocarcinoma | 49 | 340 | cg26024851 | -0.008 | -0.02 | Weak |
|  |  |  |  | cg12262564 | 0.084 | 0.004 | Weak |
|  |  |  |  | cg12441221 | 0.232 | -0.024 | Weak |
|  |  |  |  | cg17722719 | 0.078 | -0.028 | Weak |
|  |  |  |  | cg12124478 | -0.084 | 0.041 | Weak |

**Supplementary Table S7: Methylation status of IQGAP3 at promoter region and its correlation with the mRNA expression**
